# Associations between structural stigma and earlier pubertal timing persist for 1 year among Black girls and Latinx youth

**DOI:** 10.1038/s41598-025-00378-8

**Published:** 2025-05-21

**Authors:** Rachel M. Martino, Nathan L. Hollinsaid, Natalie L. Colich, Katie A. McLaughlin, Mark L. Hatzenbuehler

**Affiliations:** 1 Department of Psychology, Harvard University, William James Hall, 33 Kirkland Street, Cambridge, MA 02138 USA; 2 The Ballmer Institute for Children’s Behavioral Health, University of Oregon, Portland, OR USA

**Keywords:** Childhood and adolescence, Structural stigma, Puberty, Development, Social determinants of health, Human behaviour, Biomarkers

## Abstract

Black and Latinx youth experience earlier pubertal timing relative to their non-stigmatized peers. Research on determinants of this increased risk has largely focused on aspects of individuals (e.g., body mass index) or their proximal environment (e.g., socioeconomic status), to the exclusion of broader macro-social factors. Using 2 years of Adolescent Brain Cognitive Development Study^®^ data, we examined whether structural stigma (e.g., state-level policies, aggregated prejudicial attitudes) was associated with hormonal and physical markers of pubertal development. Baseline results documented earlier pubertal timing among Black girls (hormones) and Latinx girls and boys (youth and/or caregiver report) in states with higher (vs. lower) levels of structural racism and xenophobia, respectively. Observed associations were comparable in effect size to a well-established correlate of pubertal development, body mass index, and remained 1 year later among these stigmatized (vs. non-stigmatized) groups. Findings suggest the need to broaden the study of determinants of pubertal development to include macro-social factors.

## Introduction

Puberty represents a critical developmental period that involves the complex interplay of hormonal and other neurobiological processes in order to prepare the body for physical maturation and sexual reproduction^1^. The age of pubertal onset has decreased steadily among youth in the United States (US) and globally, with youth today beginning puberty up to 2 years earlier than youth several decades ago^2–5^. Pubertal timing refers to when children and adolescents experience pubertal onset or changes in particular indicators of pubertal development (e.g., external, physical markers) relative to their peers^6^. It is commonly quantified by regressing stages or levels of these and other pubertal indicators (e.g., pubertal hormones) on chronological age, such that higher residuals represent youth experiencing earlier pubertal timing, and thus more advanced pubertal development than peers of the same chronological age^7,8^.

Children and adolescents with stigmatized identities—including Black youth, Latinx youth, and girls generally—exhibit earlier pubertal timing relative to their non-stigmatized peers^3,9–12^. For example, Black and Latinx girls experience earlier breast development (i.e., thelarche) and menstruation (i.e., menarche) compared to White girls^9,10,13,14^. Specifically, one study found that by age 8, only 18% of White girls had entered thelarche compared to 31% of Latinx girls and 43% of Black girls^13^. Another study found that Black and Latinx girls, respectively, reached menarche 4 and 6 months earlier than White girls^9^. Likewise, Black boys experience earlier pubertal timing than White boys, including earlier pubic hair (i.e., adrenarche) and genital (i.e., gonadarche) growth^3^. Elevated levels of pubertal hormones have been observed among both Black girls and Black boys relative to their same-aged, White peers^15^. Finally, girls begin puberty up to 1.5 years earlier than boys in the US, which has primarily been attributed to biological differences^16–20^.

Earlier pubertal onset and timing has been linked to a number of adverse physical and mental health consequences^21^, such as diabetes^22^, depression and anxiety^23–25^, externalizing disorders^26^, and substance use^27^. These consequences are especially prominent among girls^23,28^ and among racially and ethnically minoritized youth^29–32^. For example, girls who experience earlier (vs. later) pubertal timing are at increased risk for breast cancer^33^, internalizing and externalizing psychopathology^26,28,34^, and early sexual activity^35^. Additionally, earlier (vs. later) breast development has been linked to more severe mental health symptoms among Latinx girls^30^ and Black girls^36^. Similarly, earlier (vs. later) pubertal onset has been associated with depressive symptoms and externalizing symptoms among Black boys^31,32^. These studies highlight the significant need to identify and intervene on determinants of earlier pubertal timing, particularly among stigmatized youth.

Research into correlates and predictors of pubertal timing among youth has overwhelmingly focused on aspects of individuals and their proximal environment, including body mass index (BMI)^37^; indicators of family socioeconomic status (SES) such as household income^38^ and caregiver educational attainment^23^; and perceived discrimination^39^ as well as other forms of threat-related adversity (e.g., childhood emotional, physical, and sexual abuse)^23,40^. This work identifies associations between many of these factors—particularly BMI and threat-related adversity—and the timing of puberty, demonstrating that they not only represent important determinants of puberty among youth generally, but also that they may partially explain well-established disparities in pubertal timing between stigmatized and non-stigmatized youth^37,39,41–44^.

Although these studies have provided important insights into individual determinants of pubertal timing, this literature has largely ignored broader contextual factors that might also contribute to earlier pubertal timing among youth, particularly stigmatized youth, despite repeated calls for such scholarship^41,45–47^. One notable exception is a recent cohort study by Acker and colleagues^48^, which documented an association between neighborhood privilege and pubertal timing among a racially and ethnically diverse cohort of girls. Specifically, this study found that girls living in neighborhoods characterized by greater concentrations of economically and racially/ethnically marginalized groups—wherein exposure to social, economic, and environmental stressors may be elevated—experienced earlier (vs. later) pubertal timing^48^. To our knowledge, this study provided one of the only tests of whether factors operating beyond the individual or interpersonal level are associated with pubertal timing.

Building on this work, the present study provides a novel framework for widening the study of determinants of earlier pubertal timing to the macro-social level by incorporating measures of structural stigma, defined by Hatzenbuehler and Link (*p*. 2) as “societal-level conditions, cultural norms, and institutional policies and practices that constrain the opportunities, resources, and wellbeing of the stigmatized”^49^. Structural stigma is a broad concept that researchers have applied to operationalize and understand distinct subtypes of structural forms of stigma targeting particular marginalized groups, including structural racism, structural xenophobia, and structural sexism, among others^50^. Exposure to structural stigma during childhood and adolescence is not only a fundamental driver of health disparities between stigmatized and non-stigmatized youth^51–56^, but it is also an important source of within-group heterogeneity in adverse developmental and psychosocial outcomes among populations of stigmatized youth^57–59^. For example, Black and Latinx youth living in US states characterized by higher (vs. lower) levels of structural racism (i.e., more prejudicial anti-Black racial attitudes) and structural xenophobia (i.e., more restrictive immigration-related policies and more negative anti-immigrant attitudes), respectively, have smaller hippocampal volume^60^ and higher levels of psychopathology^55,59^. Moreover, sexual minority (i.e., lesbian, gay, and bisexual) young adults raised in US states with higher (vs. lower) levels of structural homophobia demonstrate blunted cortisol reactivity to stress^61^.

Life history theory^62,63^ and dimensional models of childhood adversity^64–66^ propose that developmental processes such as puberty may be accelerated in environments characterized by greater threat in order to maximize the opportunity for reproduction before mortality. The precise mechanisms through which early-life stressors accelerate pubertal onset and development remain under investigation. However, these theories posit that youth’s early exposure to environments characterized by threat may alter physiological stress response systems such the hypothalamic–pituitary–adrenal (HPA) axis, which regulates systems responsible for pubertal development, including the hypothalamic–pituitary–gonadal (HPG) axis^67–69^. Notably, structural stigma has been conceptualized as a chronic form of threat-related adversity existing at the macro-social level^70–73^. Thus, informed by life history theory and research on dimensional models of childhood adversity, we sought to examine whether exposure to structural stigma—in the form of structural racism among Black youth, structural xenophobia among Latinx youth, and structural sexism among girls—was associated with earlier pubertal timing among these three stigmatized groups.

Answering this question required a novel data structure atypical of most studies on pubertal development and timing to date. First, we needed a dataset examining pubertal development among stigmatized youth—specifically, Black youth, Latinx youth, and girls—exposed to macro-social contexts varying in their levels of structural racism, xenophobia, and sexism, respectively. Second, we needed a dataset assessing multiple indicators of pubertal development (e.g., salivary hormone levels, physical markers). This data feature would permit us to examine whether associations between structural stigma and pubertal timing differ across indicators of potentially distinct pubertal processes, given evidence of relatively low correspondence between hormonal and external, physical indicators of pubertal development^6,15,74,75^. Third, the dataset would need to include measures of individual correlates of pubertal development in order to ensure that structural stigma remains associated with pubertal timing even after accounting for aspects of individuals (e.g., BMI) and of their proximal environments (e.g., family SES) that might also be associated with pubertal timing. Fourth, we needed a dataset that also included youth not holding the stigmatized identities of interest to serve as negative control groups (e.g., non-Latinx White youth for analyses with Black and Latinx youth, boys for analyses with girls) to evaluate the consistency and specificity of findings among stigmatized (vs. non-stigmatized) youth^76^. Finally, we required a longitudinal dataset in order establish the consistency of results at separate points in development.

Fortunately, a dataset meeting these requirements recently became available. Specially, the current study leveraged baseline and Year 1 (i.e., 1-year follow-up) data from the Adolescent Brain Cognitive Development (ABCD) Study^®^ to consider whether exposure to higher (vs. lower) levels of structural racism, xenophobia, and sexism was associated with earlier (vs. later) pubertal timing among Black youth, Latinx youth, and girls, respectively. The ABCD Study^®^ was uniquely situated to address this research question. It represents one of the largest multisite, longitudinal studies of child and adolescent development to date, enrolling 11,844 youth (primarily ages 9–10) at baseline from 21 sites located in 17 US states that vary substantially in their levels of structural racism, xenophobia, and sexism^60^. We focused on Black youth, Latinx youth, and girls given ample evidence of earlier pubertal timing among these populations^3,9–12^ and their considerable representation in the ABCD Study^®^ (see “Methods” for sample sizes and demographics). Herein, we use the terms “girls” and “boys” to refer to youth’s assumed gender based on caregiver reports of their birth-assigned sex, as information on youth-reported gender and gender identity was not collected at baseline of the ABCD Study^®^.

The representation of these three stigmatized groups enabled us to link their individual-level data to well-established state-level measures of structural racism, xenophobia, and sexism as proxies for the macro-social contexts surrounding Black youth, Latinx youth, and girls, respectively^60^. Manifestations of structural racism, xenophobia, and sexism captured in these measures, respectively, comprise: (1) multiple indicators of explicit anti-Black racial prejudice aggregated to the state level; (2) two single-item indicators of explicit social attitudes towards immigrants and Latinx people aggregated to the state level as well as a composite index of state-level anti-immigration laws/policies; and (3) multiple state-level indicators of women’s social, political, and economic autonomy (e.g., women’s labor force participation) as well as implicit and explicit attitudes towards gender roles and women’s social status aggregated to the state level.

In order to capture the distinct development processes implicated in puberty^6,15,74,75,77^, we examined salivary levels of three pubertal hormones (i.e., estradiol among girls; DHEA and testosterone among both girls and boys) as well as youth- and caregiver-reported external, physical markers of pubertal development as outcomes. To index pubertal timing, these variables were regressed on chronological age, with higher (i.e., more positive) residuals corresponding to youth experiencing earlier pubertal timing, and thus more advanced pubertal development on these indicators than peers of the same chronological age^7,8^. In preregistered hypotheses informed by life history theory^62,63^ and dimensional models of childhood adversity^64–66^—as well as by prior research on associations between structural stigma and developmental and psychosocial outcomes in childhood and adolescence^55,59–61^—we predicted that Black youth, Latinx youth, and girls living in ABCD Study^®^ states characterized by higher (vs. lower) levels of structural racism, xenophobia, and sexism, respectively, would evidence earlier pubertal timing at baseline and Year 1. We also sought to establish whether (or not) these structural stigma measures were associated with pubertal timing among non-stigmatized comparison groups (i.e., non-Latinx White girls and boys for primary analyses with Black and Latinx girls and boys, boys for primary analyses with girls) as a form of negative control analysis.

## Results

In preregistered analyses, we used linear mixed-effects models to examine associations between structural racism, xenophobia, and sexism—quantified objectively using existing measures of state-level policies, aggregated prejudicial attitudes, and/or other societal conditions^60^—and hormonal indicators as well as youth- and caregiver-reported external, physical markers of pubertal development among Black youth, Latinx youth, and girls, respectively. These indicators of pubertal development were regressed on chronological age to index pubertal timing, and relevant analyses were stratified by birth-assigned sex to account for potential biological differences in pubertal onset and development^16–20^. As we expected relatively modest changes in our study’s measures of pubertal development over the course of just 1 year of late childhood/early adolescence^78^, we preregistered repeated cross-sectional (vs. longitudinal) tests of these associations at baseline and Year 1 of the ABCD Study^®^, enabling us to assess the consistency of results across these two timepoints. We controlled for BMI, family SES in the form of mean caregiver educational attainment, and another structural factor—state-level income inequality—in order to evaluate whether structural racism, xenophobia, and sexism remained associated with pubertal timing over and above individual and proximal correlates of pubertal development as well as a broader form of social inequality. Finally, as a form of negative control analysis^76^, we reran our primary models among non-stigmatized comparison groups (i.e., non-Latinx White girls and boys for primary analyses conducted with Black and Latinx girls and boys, boys for primary analyses conducted with girls), allowing us to evaluate whether and how consistently associations between structural racism, xenophobia, and sexism and pubertal timing were observed only among stigmatized (vs. non-stigmatized) youth.

### Associations between structural stigma and pubertal timing using hormonal indicators of pubertal development

#### Structural racism and earlier (vs. later) pubertal timing using hormonal indicators of pubertal development among Black and non-Latinx White girls

**Fig. 1:**
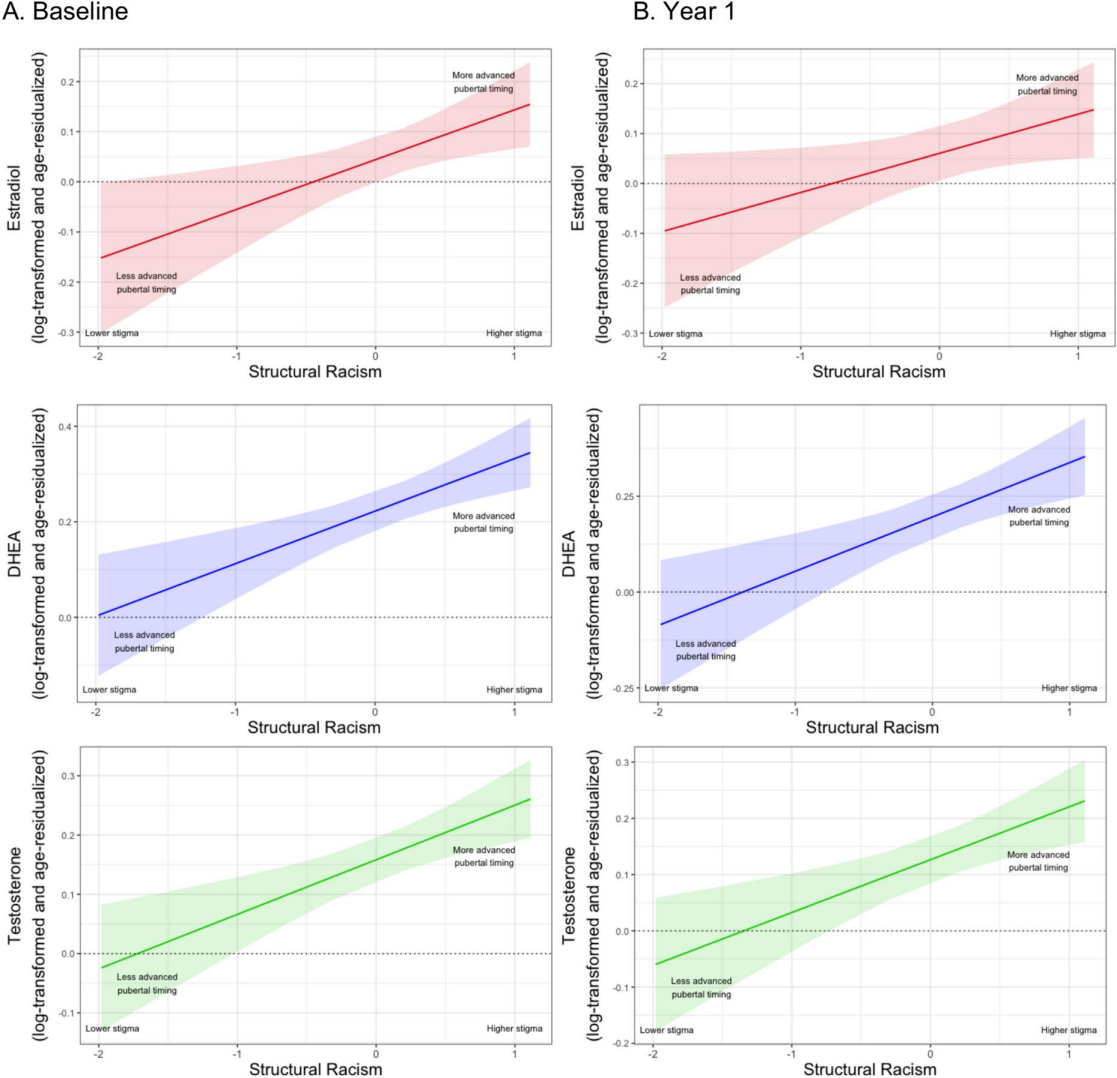
Associations between structural racism and pubertal timing using hormonal indicators of pubertal development among Black girls. Note: Panel A shows significant associations between structural racism and pubertal timing via three hormonal indicators of pubertal development among Black girls at baseline: estradiol (*p* = 0.006**), DHEA (*p* < 0.001***), and testosterone (*p* < 0.001***). Panel B shows that the associations between structural racism and pubertal timing via these three hormonal indicators remained significant among Black girls at Year 1: estradiol (*p* = 0.033*), DHEA (*p* < 0.001***) and testosterone (*p* < 0.001***). *p < 0.05, **p < 0.01, ***p < 0.001.

*Primary analysis with Black girls.* At baseline, Black girls living in ABCD Study^®^ states characterized by higher (vs. lower) levels of structural racism experienced earlier pubertal timing via all three hormonal indicators (Fig. 1A, Table 1): estradiol (β = 0.12, 95% CI [0.04, 0.20], *p* = 0.006); DHEA (β = 0.11, 95% CI [0.05, 0.17], *p* < 0.001); and testosterone (β = 0.13, 95% CI [0.06, 0.20], *p* < 0.001). At Year 1, we continued to observe significant associations between structural racism and earlier pubertal timing via these three hormonal indicators among Black girls (Fig. 1B, Table 1): estradiol (β = 0.10, 95% CI [0.01, 0.19], *p* = 0.033); DHEA (β = 0.14, 95% CI [0.06, 0.22], *p* < 0.001); and testosterone (β = 0.14, 95% CI [0.06, 0.23], *p* < 0.001). These associations remained significant when excluding Black girls who experienced menarche, suggesting that findings were not simply driven by post-menarche changes in pubertal hormones (Supplementary Table 1).

**Table 1:** Associations between structural racism and pubertal timing using hormonal indicators of pubertal development among Black girls.

	Baseline						Year 1					
	b	SE	*z*	*p*	*β*	95% CI	b	SE	*z*	*p*	*β*	95% CI
Associations between structural racism and earlier (vs. later) pubertal timing using hormonal indicators of pubertal development among Black girls: Estradiol												
Intercept	− 1.303	0.607	− 2.147	0.032*	0.002	(− 0.073, 0.078)	− 1.186	0.715	− 1.659	0.097	− 0.014	(− 0.111, 0.083)
Structural racism	0.099	0.036	2.775	0.006**	0.118	(0.035, 0.202)	0.079	0.037	2.128	0.033*	0.098	(0.008, 0.188)
BMI	0.014	0.004	3.675	< 0.001***	0.112	(0.052, 0.171)	0.013	0.004	3.275	0.001**	0.112	(0.045, 0.180)
Caregiver education	0.015	0.007	2.086	0.037*	0.066	(0.004, 0.128)	− 0.003	0.008	− 0.380	0.704	− 0.014	(− 0.085, 0.058)
State income inequality	1.226	1.227	0.999	0.318	0.037	(− 0.035, 0.108)	1.440	1.446	0.995	0.320	0.045	(− 0.043, 0.132)
Collection time	0.020	0.006	3.304	< 0.001***	0.105	(0.043, 0.167)	0.023	0.006	3.611	< 0.001***	0.131	(0.060, 0.202)
Associations between structural racism and earlier (vs. later) pubertal timing using hormonal indicators of pubertal development among Black girls: DHEA												
Intercept	− 0.038	0.602	− 0.063	0.950	− 0.003	(− 0.062, 0.056)	− 0.900	0.789	− 1.140	0.254	− 0.006	(− 0.087, 0.074)
Structural racism	0.110	0.030	3.673	< 0.001***	0.111	(0.052, 0.170)	0.142	0.040	3.545	< 0.001***	0.140	(0.063, 0.217)
BMI	0.034	0.004	7.959	< 0.001***	0.231	(0.174, 0.287)	0.035	0.005	6.850	< 0.001***	0.229	(0.164, 0.295)
Caregiver education	0.002	0.008	0.245	0.807	0.007	(− 0.052, 0.067)	0.001	0.010	0.102	0.919	0.004	(− 0.065, 0.072)
State income inequality	− 0.485	1.216	− 0.399	0.690	− 0.012	(− 0.071, 0.047)	0.782	1.593	0.491	0.624	0.019	(− 0.057, 0.096)
Collection time	− 0.019	0.006	− 3.014	0.003**	− 0.087	(− 0.143, − 0.030)	− 0.003	0.008	− 0.349	0.727	− 0.012	(− 0.082, 0.057)
Associations between structural racism and earlier (vs. later) pubertal timing using hormonal indicators of pubertal development among Black girls: Testosterone												
Intercept	0.350	0.521	0.672	0.502	− 0.011	(− 0.086, 0.064)	− 0.359	0.552	− 0.651	0.515	− 0.010	(− 0.098, 0.078)
Structural racism	0.092	0.026	3.589	< 0.001***	0.130	(0.059, 0.200)	0.094	0.028	3.306	< 0.001***	0.141	(0.057, 0.225)
BMI	0.016	0.003	5.070	< 0.001***	0.150	(0.092, 0.208)	0.014	0.003	4.005	< 0.001***	0.137	(0.070, 0.203)
Caregiver education	− 0.007	0.006	− 1.045	0.296	− 0.033	(− 0.095, 0.029)	− 0.003	0.007	− 0.520	0.603	− 0.019	(− 0.088, 0.051)
State income inequality	− 0.468	1.036	− 0.451	0.652	− 0.016	(− 0.088, 0.055)	0.619	1.115	0.555	0.579	0.023	(− 0.059, 0.105)
Collection time	− 0.016	0.005	− 3.045	0.002**	− 0.098	(− 0.161, − 0.035)	− 0.004	0.006	− 0.644	0.520	− 0.024	(− 0.097, 0.049)

Hormone levels of estradiol, DHEA, and testosterone were log-transformed and age-residualized to index earlier (vs. later) pubertal timing.

BMI, body-mass index.

*p < 0.05, **p < 0.01, ***p < 0.001.

*Negative control analysis with non-Latinx White girls*. We also found significant baseline associations between structural racism and earlier pubertal timing via two hormonal indicators among non-Latinx White girls: DHEA (β = 0.07, 95% CI [0.01, 0.13], *p* = 0.032) and testosterone (β = 0.11, 95% CI [0.02, 0.19], *p* = 0.013). However, these associations were relatively smaller in magnitude among non-Latinx White (vs. Black) girls, and no such association was observed for the third hormonal indicator, estradiol, among non-Latinx White girls (Supplementary Table 2). Moreover, no significant associations between structural racism and pubertal timing were observed for any of these hormonal indicators among non-Latinx White girls at Year 1 (Supplementary Table 2). Thus, results from these negative control analyses provide suggestive evidence that structural racism was most consistently (i.e., for all three pubertal hormones) and persistently (i.e., at baseline and Year 1) associated with earlier pubertal timing via hormonal indicators of pubertal development among Black (vs. non-Latinx White) girls.

#### Structural stigma and earlier (vs. later) pubertal timing using hormonal indicators of pubertal development among Black boys, Latinx youth, and girls

*Primary analysis with Black boys, Latinx youth, and girls*. No significant associations between structural stigma and pubertal timing via hormonal indicators of pubertal development were documented among the other stigmatized groups in our study, including for structural racism among Black boys, structural xenophobia among Latinx girls and boys, and structural sexism among girls (Supplementary Tables 3–6).

### Associations between structural stigma and pubertal timing using external, physical markers of pubertal development

#### Structural xenophobia and earlier (vs. later) pubertal timing using external, physical markers of pubertal development among Latinx and non-Latinx White youth

**Fig. 2:**
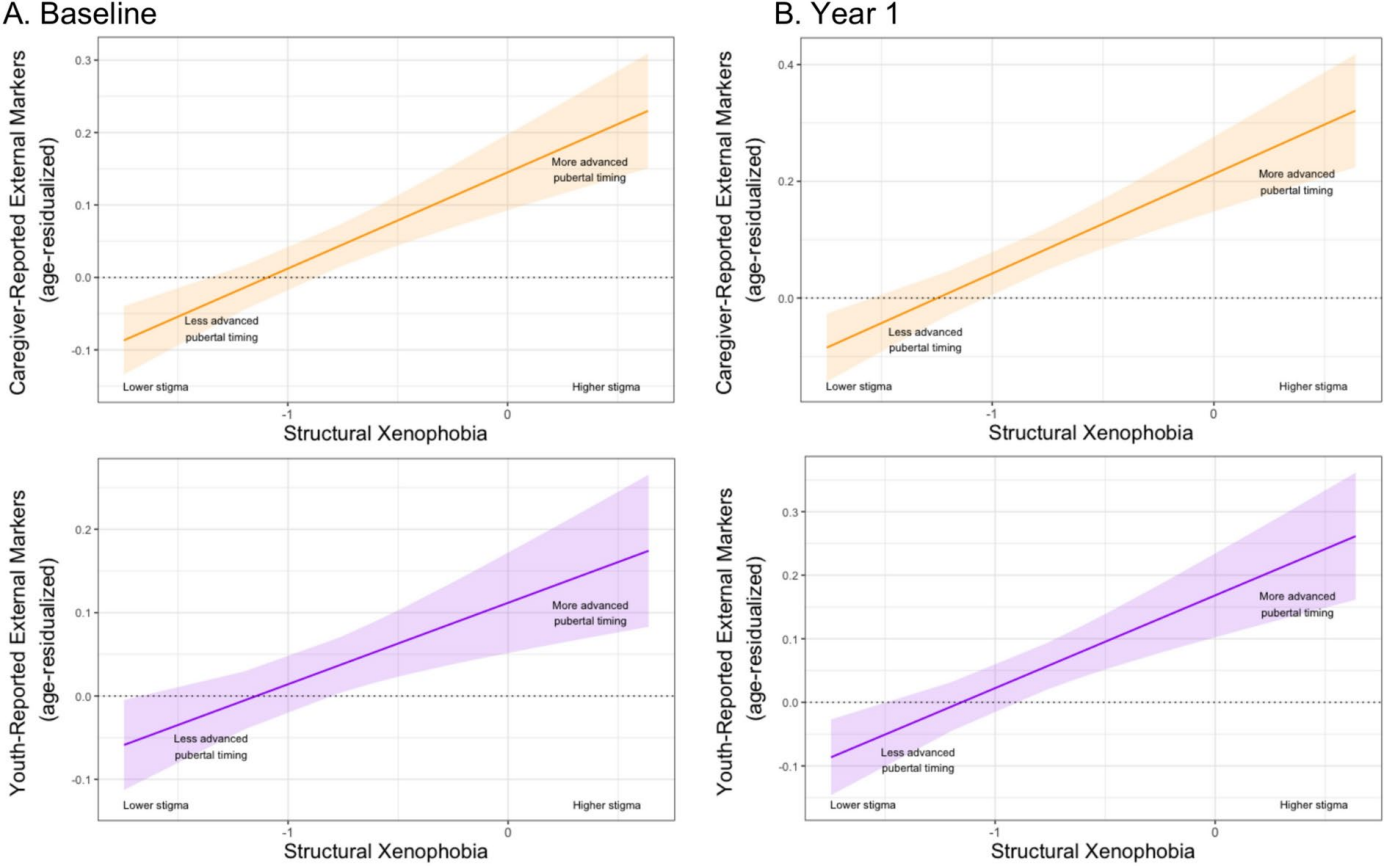
Associations between structural xenophobia and pubertal timing using caregiver- and youth-reported external, physical markers of pubertal development among Latinx girls. Note: Panel A shows significant associations between structural xenophobia and pubertal timing via both caregiver-reported external, physical markers (*p* < 0.001***) and youth-reported external, physical markers (*p* < 0.001***) of pubertal development among Latinx girls at baseline. Panel B shows that associations between structural xenophobia and pubertal timing via caregiver-reported external, physical markers (*p* < 0.001***) and youth-reported external, physical markers (*p* < 0.001***) of pubertal development remained significant among Latinx girls at Year 1. *p < 0.05, **p < 0.01, ***p < 0.001.

*Primary analysis with Latinx girls*. At baseline, Latinx girls (β = 0.19, 95% CI [0.12, 0.26, *p* < 0.001; Fig. 2A) living in US states with higher (vs. lower) levels of structural xenophobia experienced earlier pubertal timing via caregiver-reported external, physical markers of pubertal development (Table 2). This association remained significant for Latinx girls (β = 0.21, 95% CI [0.14, 0.28], *p* < 0.001; Fig. 2B) at Year 1 (Table 2). Likewise, Latinx girls living in states with higher (vs. lower) levels of structural xenophobia also experienced earlier pubertal timing via youth-reported external, physical markers of pubertal development at baseline (β = 0.13, 95% CI [0.06, 0.20], *p* < 0.001; Fig. 2A) and Year 1 (β = 0.18, 95% CI [0.11, 0.25], *p* < 0.001; Fig. 2B; see also Table 3). In preregistered supplemental analyses, a similar pattern of results was observed when examining pubertal timing using alternative specifications of these indicators (i.e., external, physical markers of adrenarche and gonadarche, pubertal development categories). These additional analyses provided generally consistent evidence of earlier (vs. later) pubertal timing among Latinx girls in more (vs. less) xenophobic states—both with respect to caregiver- and youth-reported pubertal development categories and to caregiver- and youth-reported external, physical markers of adrenarche and gonadarche (Supplementary Tables 7–10).

**Table 2:** Associations between structural xenophobia and pubertal timing using caregiver-reported external, physical markers of pubertal development among Latinx girls and boys.

	Baseline						Year 1					
	b	SE	*z*	*p*	*β*	95% CI	b	SE	*z*	*p*	*β*	95% CI
Associations between structural xenophobia and earlier (vs. later) pubertal timing using caregiver-reported external, physical markers of pubertal development among Latinx girls												
Intercept	− 2.420	0.426	− 5.679	< 0.001***	0.004	(− 0.051, 0.060)	− 2.645	0.526	− 5.025	< 0.001***	0.004	(− 0.054, 0.062)
Structural xenophobia	0.133	0.024	5.533	< 0.001***	0.193	(0.124, 0.261)	0.170	0.029	5.785	< 0.001***	0.211	(0.140, 0.283)
BMI	0.034	0.004	9.385	< 0.001***	0.270	(0.214, 0.326)	0.039	0.004	9.493	< 0.001***	0.286	(0.227, 0.345)
Caregiver education	0.000	0.004	0.033	0.974	0.001	(− 0.057, 0.059)	0.002	0.005	0.352	0.725	0.011	(− 0.049, 0.071)
State income inequality	3.952	0.895	4.414	< 0.001***	0.153	(0.085, 0.221)	4.207	1.106	3.804	< 0.001***	0.138	(0.067, 0.209)
Associations between structural xenophobia and earlier (vs. later) pubertal timing using caregiver-reported external, physical markers of pubertal development among Latinx boys												
Intercept	− 1.236	0.370	− 3.345	< 0.001***	0.010	(− 0.047, 0.066)	− 1.674	0.425	− 3.939	< 0.001***	0.004	(− 0.055, 0.062)
Structural xenophobia	0.054	0.019	2.798	0.005**	0.097	(0.029, 0.165)	0.058	0.022	2.603	0.009**	0.095	(0.024, 0.167)
BMI	0.014	0.003	5.209	< 0.001***	0.146	(0.091, 0.200)	0.017	0.003	5.555	< 0.001***	0.163	(0.106, 0.221)
Caregiver education	− 0.009	0.004	− 2.404	0.016*	− 0.071	(− 0.129, − 0.013)	0.005	0.004	1.175	0.24	0.036	(− 0.024, 0.097)
State income inequality	2.482	0.772	3.216	< 0.0001**	0.112	(0.044, 0.180)	2.843	0.889	3.198	< 0.001***	0.117	(0.045, 0.189)

Caregiver-reported external, physical markers of pubertal development on the Pubertal Development Scale were averaged and age-residualized to index earlier (vs. later) pubertal timing.

BMI, body-mass index.

*p < 0.05, **p < 0.01, ***p < 0.001.

**Table 3:** Associations between structural xenophobia and pubertal timing using youth-reported external, physical markers of pubertal development among Latinx girls and boys.

	Baseline						Year 1					
	b	SE	*z*	*p*	*β*	95% CI	b	SE	*z*	*p*	*β*	95% CI
Associations between structural xenophobia and earlier (vs. later) pubertal timing using youth-reported external, physical markers of pubertal development among Latinx girls												
Intercept	− 1.688	0.492	− 3.432	< 0.001***	0.001	(− 0.056, 0.058)	− 2.206	0.541	− 4.078	< 0.001***	0.005	(− 0.055, 0.064)
Structural xenophobia	0.098	0.028	3.539	< 0.001***	0.126	(0.056, 0.196)	0.146	0.030	4.814	< 0.001***	0.180	(0.107, 0.253)
BMI	0.025	0.004	6.049	< 0.001***	0.181	(0.123, 0.240)	0.029	0.004	6.832	< 0.001***	0.211	(0.151, 0.272)
Caregiver education	0.004	0.005	0.882	0.378	0.027	(− 0.033, 0.086)	0.004	0.005	0.701	0.483	0.022	(− 0.040, 0.084)
State income inequality	2.570	1.031	2.493	0.013*	0.088	(0.019, 0.158)	3.577	1.136	3.148	0.002**	0.117	(0.044, 0.190)
Associations between structural xenophobia and earlier (vs. later) pubertal timing using youth-reported external, physical markers of pubertal development among Latinx boys												
Intercept	− 0.540	0.651	− 0.830	0.406	− 0.014	(− 0.104, 0.076)	− 1.239	0.650	− 1.906	0.057	− 0.029	(− 0.137, 0.079)
Structural xenophobia	0.029	0.043	0.678	0.498	0.039	(− 0.073, 0.151)	0.091	0.044	2.050	0.040*	0.122	(0.005, 0.238)
BMI	0.004	0.004	1.008	0.314	0.029	(− 0.027, 0.085)	0.010	0.004	2.760	0.006**	0.083	(0.024, 0.141)
Caregiver education	− 0.013	0.005	− 2.635	0.008**	− 0.078	(− 0.136, − 0.020)	− 0.004	0.005	− 0.736	0.462	− 0.023	(− 0.083, 0.038)
State income inequality	1.481	1.400	1.058	0.290	0.050	(− 0.042, 0.142)	2.587	1.389	1.863	0.063	0.088	(− 0.005, 0.180)

Youth-reported external, physical markers of pubertal development on the Pubertal Development Scale were averaged and age-residualized to index earlier (vs. later) pubertal timing.

BMI, body-mass index.

*p < 0.05, **p < 0.01, ***p < 0.001.

**Fig. 3:**
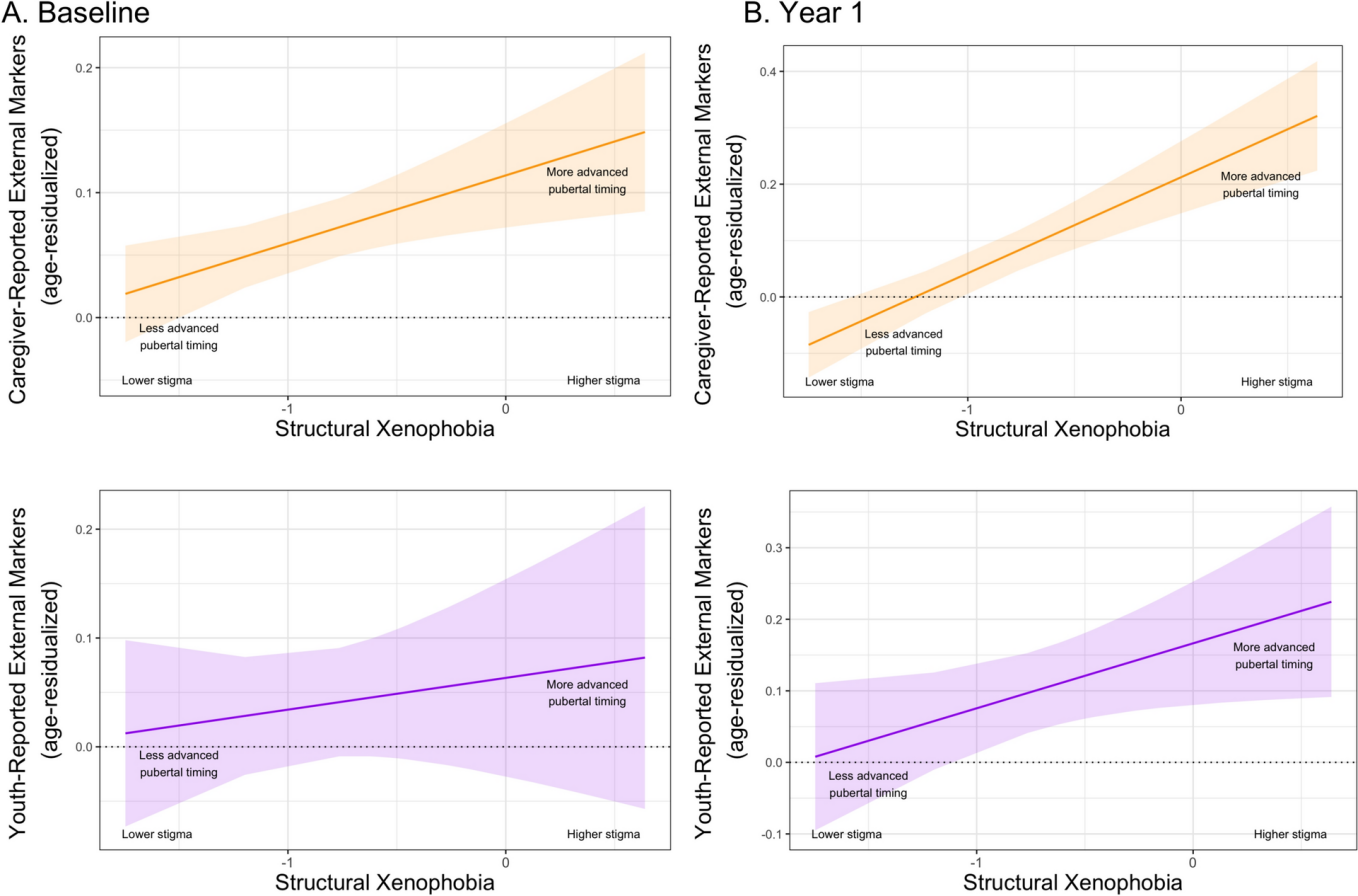
Associations between structural xenophobia and pubertal timing using caregiver- and youth-reported external, physical markers of pubertal development among Latinx boys. Note: Panel A shows a significant association between structural xenophobia and pubertal timing via caregiver-reported external, physical markers (*p* = 0.005**) of pubertal development among Latinx boys at baseline. Although structural xenophobia was not significantly associated with pubertal timing via youth-reported external, physical markers of pubertal development among Latinx boys at baseline, Panel B shows that structural xenophobia was significantly associated with pubertal timing via both caregiver-reported external, physical markers (*p* = 0.009**) and youth-reported external, physical markers (*p* = 0.040*) of pubertal development among Latinx boys at Year 1. *p < 0.05, **p < 0.01, ***p < 0.001.

*Primary analysis with Latinx boys*. In US states with higher (vs. lower) levels of structural xenophobia, Latinx boys experienced earlier pubertal timing via caregiver-reported external, physical markers of pubertal development at baseline (β = 0.10, 95% CI [0.03, 0.17], *p* = 0.005; Fig. 3A) and at Year 1 (β = 0.10, 95% CI [0.02, 0.17], *p* = 0.009; Fig. 3B; Table 2). Although no association between structural xenophobia and pubertal timing via youth-reported external, physical markers of pubertal development was observed among Latinx boys at baseline (Fig. 3A), a significant association emerged among Latinx boys at Year 1 (β = 0.12, 95% CI [0.01, 0.24], *p* = 0.040), with Latinx boys living in US states with higher (vs. lower) structural xenophobia experiencing earlier pubertal timing via this indicator (Fig. 3B; Table 3). Supplemental analyses revealed generally consistent results at Year 1 when examining earlier (vs. later) pubertal timing among Latinx boys, with respect both to caregiver- and youth-reported pubertal development categories and to caregiver- and youth-reported external, physical markers of adrenarche and gonadarche (Supplementary Tables 11–14).

*Negative control analysis with non-Latinx girls and boys.* Although some significant associations between structural xenophobia and earlier pubertal timing via caregiver- or youth-reported external, physical markers of pubertal development were observed among non-Latinx White girls and boys at baseline, no significant associations were found among non-Latinx White girls and boys at Year 1 (Supplementary Tables 15–26). Taken together with findings from our primary analyses with Latinx girls and boys, results from these negative control analyses suggest that structural xenophobia was more consistently associated with earlier pubertal timing related to external, physical markers of pubertal development among Latinx (vs. non-Latinx White) youth—and most persistently so for Latinx girls.

#### Structural stigma and earlier (vs. later) pubertal timing using external, physical markers of pubertal development among Black youth and girls

*Primary Analysis with Black Youth and Girls.* No associations between structural stigma and puberal timing indexed via caregiver- and youth-reported external, physical markers of pubertal development were observed among the other stigmatized groups in our study, including for structural racism among Black girls and boys and for structural sexism among girls (Supplementary Tables 27–32).

## Discussion

Youth with stigmatized identities, including racially (e.g., Black) and ethnically (e.g., Latinx) minoritized youth as well as girls, experience earlier pubertal onset and timing relative to their non-stigmatized peers^9,11–13,20,79,80^. Research into correlates of this increased risk has largely focused on aspects of individuals and of their proximal environments^37–39,42,44,81,82^, despite repeated calls to consider broader contextual factors operating at the macro-social level^41,45,47^. Leveraging 2 years of ABCD Study^®^ data, we provide novel evidence that one such factor—exposure to structural stigma at the state level—is associated with earlier (vs. later) pubertal timing among multiple stigmatized groups, including Black girls and Latinx youth.

Specifically, baseline results demonstrated that Black girls living in states characterized by higher (vs. lower) structural racism experienced earlier pubertal timing indexed via three hormonal indicators: estradiol, DHEA, and testosterone. At baseline, we also documented evidence of earlier pubertal timing via caregiver- and youth-reported external, physical markers of pubertal development among Latinx girls, as well as via caregiver-reported external, physical markers of pubertal development among Latinx boys, in states with higher (vs. lower) structural xenophobia. These significant cross-sectional associations between structural racism and xenophobia and earlier pubertal timing among Black girls and Latinx youth, respectively, persisted 1 year later. In addition, a significant association between structural xenophobia and earlier pubertal timing via youth-reported external, physical markers of pubertal development emerged among Latinx boys at Year 1. Significant associations between structural racism and xenophobia and earlier pubertal timing among Black girls and Latinx youth, respectively, were generally comparable in effect size to associations between BMI and pubertal timing in our models. This finding suggests that structural racism and xenophobia, although operating at a more distal level, may have a comparable influence on earlier (vs. later) pubertal timing to BMI, one of the most widely studied and robust predictors of early pubertal onset and advanced pubertal development^19,82–84^. Although structural racism and xenophobia were also associated with some indicators of pubertal timing among non-Latinx White girls and boys at baseline, no such associations were observed among these non-stigmatized comparison groups at Year 1. By comparison, significant associations between structural racism and xenophobia and earlier pubertal timing were persistently observed, respectively, among Black girls (for salivary levels of three pubertal hormones) and Latinx youth (for caregiver- and/or youth-reported external, physical markers of pubertal development), providing some evidence of the consistency and specificity of these findings among these two stigmatized (vs. non-stigmatized) groups.

In sum, we observed significant associations between measures of structural stigma and one or more indicators of early pubertal timing among two of the three stigmatized groups in our study, which either persisted from baseline to Year 1 or emerged at Year 1 only among stigmatized (vs. non-stigmatized) youth. These findings have several important implications. First, they suggest that Black girls and Latinx youth living in US states characterized by higher (vs. lower) levels of structural racism and xenophobia, respectively, may begin puberty earlier or experience puberty faster than their same-aged peers, which would provide a plausible explanation for our observations of earlier (vs. later) pubertal timing at baseline and Year 1 among Black girls (via three pubertal hormones) and Latinx youth (via caregiver- and/or youth-reported external, physical markers) in higher-stigma contexts. As significant proportions of Black girls and Latinx girls and boys reside in US states ranking above the national average on our study’s measures of structural racism and xenophobia^85^, their exposure to more (vs. less) stigmatizing environments may partially contribute to the earlier ages of pubertal onset and development consistently documented among these stigmatized groups relative to their non-stigmatized peers^4,9,10,13,20,80,86^.

Second, our study’s structural racism and xenophobia measures exclusively or largely captured indicators characteristic of threatening macro-social environments—including aggregated anti-Black racial prejudice and aggregated explicit attitudes towards immigrants and Latinx people, respectively. Accordingly, evidence of earlier pubertal timing among Black girls and Latinx youth in states with higher levels of structural stigma on these two measures provides support for life history theory^62,63^ and dimensional models of childhood adversity^64–66^ at the macro-social level. In line with these theoretical frameworks, just as early exposure to threat-related social adversity has been shown to accelerate puberty^23,40,67^, it is possible that macro-social environments explicitly threatening the rights, safety, and wellbeing of the stigmatized may similarly advance puberty among stigmatized youth.

Third, because significant baseline associations between structural racism and xenophobia and earlier (vs. later) pubertal timing among Black girls and Latinx girls and boys, when observed, remained significant 1 year later, it is possible that these youth may experience more persistent risk for a range of chronic mental and physical health consequences linked to earlier pubertal timing^21,23,24,87–90^.

Fourth, evidence of significant associations between structural racism and xenophobia and separate indicators of earlier pubertal timing among these two stigmatized groups—namely, via pubertal hormones among Black girls but via external, physical markers of puberty among Latinx girls and boys—may suggest that the unique macro-social contexts surrounding these groups influence interrelated yet distinct aspects of pubertal development captured by our study’s measures of these constructs^15,74,75^. Future research is needed to clarify the neuroendocrine pathways underlying these oftentimes discordant indicators of pubertal development^15,75,77,91–94^ and to determine why exposure to distinct manifestations of structural stigma might activate different pubertal processes—as may also be the case for other forms of social adversity^42,95^.

Contrary to our expectations, we did not observe significant associations between structural sexism and pubertal timing among girls. Whereas our study’s measures of structural racism and xenophobia primarily included state-level policies and/or aggregated prejudicial attitudes, the structural sexism measure also incorporated indicators of other societal conditions, including women’s access to social, economic, educational, and political resources at the state level. The inclusion of these indicators might explain the lack of observed associations between structural sexism and pubertal timing among girls in a few ways. First, these indicators of structural sexism may better reflect macro-social contexts characterized by material deprivation (vs. social threat), wherein diminished access to resources may be less conducive to reproduction, and thus delay the onset of puberty^7,23^. Second, although these and similar indicators of structural sexism have been associated with adverse health outcomes among adult women^96,97^, they may be less relevant to early adolescent girls in the ABCD Study^®^. Indeed, prior studies have not reliably documented associations between our study’s measure of structural sexism and adverse developmental or psychosocial outcomes among girls during late childhood and early adolescence^55,60^. Future research is needed to evaluate these possibilities and to test the generalizability of our findings across other operationalizations of structural sexism.

Whereas significant associations between structural stigma and earlier pubertal timing were found among Black and Latinx girls at both baseline and Year 1, these associations were not observed among Black boys and were somewhat less consistently observed among Latinx boys. This pattern of findings may be attributable to documented differences in pubertal onset and development between girls and boys, with puberty typically beginning later among boys^6,19,20^. Accordingly, associations between structural racism and xenophobia and earlier pubertal timing among Black and Latinx boys, respectively, may emerge more reliably later in adolescence, which can be examined as additional waves of ABCD Study^®^ data become available. Future research should also examine whether specific sociocultural factors or practices might moderate associations between structural stigma and pubertal timing, particularly among Black boys. For example, there is some evidence that particular racial socialization practices designed to prepare Black youth for bias and discrimination may be more protective for Black boys than girls^98–100^. This or other sociocultural moderators may help to explain why structural racism was not associated with pubertal timing among Black boys in the present study.

Study findings provide converging support for a small but growing number of studies documenting associations between exposure to structural stigma and altered developmental and adverse psychosocial outcomes—including elevated psychopathology^55,57,59^, smaller hippocampal volume^60^, and dysregulated cortisol reactivity to stress^61^—among stigmatized youth. Taken together with results from our study, these findings provide suggestive evidence that structural stigma exposure during childhood and adolescence may interfere with puberty as well as a range of other developmental and psychosocial processes, particularly among stigmatized youth. Future research is needed to identify specific mechanisms through which structural stigma might confer such risks. With respect to pubertal timing, the stress associated with early and ongoing threat in the form of structural stigma may alter HPA axis reactivity, activating the HPG axis to accelerate pubertal onset and development^67–69^. More broadly, elevated corticotropin-releasing hormone, which has been linked to altered pubertal and hippocampal development in animals^101–103^, may represent a pluripotent mechanism through which the chronic stress or lack of social safety associated with structural stigma exposure affects puberty and other developmental outcomes^60,72^.

A puzzling question is why baseline associations between structural racism and xenophobia and earlier pubertal timing were occasionally observed among non-stigmatized comparison groups, particularly non-Latinx White girls. Although unexpected, such a finding is not entirely without precedent in the literature. For example, prior work has documented associations between structural racism and negative health outcomes among Black and White populations in the US^104^. One potential explanation is that structural racism and xenophobia manifest as diminished social cohesion or opposition to social policies and programs perceived by the non-stigmatized as disproportionately benefitting the stigmatized, ultimately resulting in state-level sociopolitical environments that are detrimental to healthy developmental outcomes, including perhaps early pubertal timing, among both stigmatized and non-stigmatized youth^105–107^. Relatedly, because structural racism and xenophobia are rooted in systems that uphold and perpetuate the dominance of White, cisgender men^108,109^, measures of these constructs may also be associated with pubertal timing among non-Latinx White girls. A third possibility is that these two structural stigma measures were correlated with an unmeasured contextual variable associated with earlier (vs. later) pubertal timing among youth generally. However, this explanation is less likely, as our primary results were robust to one such contextual factor: state-level income inequality. Perhaps the most plausible explanation is that the considerably larger sample of non-Latinx White (vs. Black and Latinx) girls and boys, particularly at baseline, provided substantially greater statistical power to detect relatively weak yet significant associations between measures of structural racism and xenophobia and pubertal timing among non-stigmatized comparison groups at this timepoint. Future studies with more equitable representation between stigmatized and non-stigmatized youth are needed to evaluate these possibilities.

Our study has several notable methodological strengths that can guide future research into macro-social correlates of pubertal development, including earlier pubertal timing. First, we linked objective measures of structural stigma to individual-level data from ABCD Study^®^ youth living in 17 US states, offering unprecedented tests of associations between structural racism, xenophobia, and sexism and pubertal timing among Black youth, Latinx youth, and girls, respectively. Second, we tested associations between these structural stigma measures and pubertal timing indexed via multiple indicators—including salivary levels of three pubertal hormones as well as caregiver- and youth-reported external, physical markers of pubertal development at baseline and Year 1—enabling us to evaluate the consistency of findings across measures, informants, and time. Third, we controlled for BMI, family SES in the form of mean caregiver educational attainment, and state-level income inequality, demonstrating that our results were robust to two established individual correlates of pubertal timing and to a broader feature of youth’s macro-social environment. Fourth, we ran negative control analyses with non-stigmatized comparators, providing evidence that significant associations between structural racism and xenophobia and earlier pubertal timing, when observed at baseline, persisted at Year 1 only among stigmatized youth—and most consistently so for Black and Latinx girls.

These strengths notwithstanding, there are several study limitations that might also inform future research on structural stigma and pubertal timing. First, although the ABCD Study^®^ represents one of the largest investigations into child and adolescent development to date, findings may not generalize to youth from US states not included in this study. To the extent that the exclusion of some US states limited variability in ABCD Study^®^ youth’s exposure to structural stigma, however, our findings are likely conservative. Future studies with even greater variability in exposure to structural stigma are needed to examine this possibility. Our focus on structural stigma at the state level was warranted given ABCD Study^®^ youth’s differential exposure to US states that varied considerably in levels of structural racism, xenophobia, and sexism. However, future studies would benefit from incorporating measures of structural stigma at more proximal geographic levels (e.g., schools, cities, counties), which would not only account for within-state variability in pubertal development but might also exert a stronger influence on pubertal timing, as has been shown for psychosocial outcomes (e.g., identity concealment)^110^. If that is the case, then our study once again provides relatively conservative tests of associations between structural stigma and pubertal timing. Additionally, we were unable to account for potential geographic mobility between states from baseline to Year 1. To the extent that mobility occurred, we would generally expect it to be non-differential by pubertal timing and structural stigma context, which would introduce random measurement error rather than systematic bias. Although it is plausible that families may have moved from higher-stigma to lower-stigma states to escape hostile macro-social environments, relocation in this direction would likely bias our findings towards the null.

Second, all three structural stigma measures included indicators of individual prejudicial attitudes aggregated to the state level and pooled across available years. This approach offers several benefits, including that it reduces measurement error by ensuring that there are sufficient observations for each US state and that it comprises a range of years overlapping with the life course of ABCD Study^®^ youth, thereby approximating the macro-social contexts surrounding these youth across development^60^. At the same time, this aggregation method may not account for temporal variability in implicit and explicit social attitudes, which have become less biased towards women and racially and ethnically minoritized groups in the US in recent years^111^. However, there is evidence that relative rankings of aggregated prejudicial attitudes towards these groups have remained generally stable between US states over this period of time^112–114^, supporting the validity of a time-invariant approach. Nevertheless, future studies should take advantage of emerging methods, such as natural language processing of media and language corpora^115–117^, which may enable scholars to develop time-variant structural stigma measures, and thus pinpoint when during the life course structural stigma is most consequential to developmental outcomes such as puberty.

Third, although we examined associations between structural stigma and pubertal timing using multiple indicators of pubertal development at baseline and Year 1 of the ABCD Study^®^, these analyses were cross-sectional. We preregistered cross-sectional (rather than longitudinal) analyses at baseline and Year 1 because detecting what we expected to be relatively modest changes in our study’s measures of pubertal development over the course of just 1 year of late childhood/early adolescence would have required considerable statistical power^78^, particularly if baseline levels of pubertal timing were also associated with structural stigma, as we hypothesized. Although we cannot infer causality from repeated cross-sectional analyses, we can be confident in ruling out reverse causation as an explanation for our findings because earlier pubertal timing at the individual level would not be expected to influence social policies, attitudes, and conditions at the societal level. Future research can enhance casual inferences by using longitudinal designs that repeatedly assess hormonal and external, physical indicators of pubertal development over much longer periods of time, which would also enable tests of whether structural stigma is associated with the pace of pubertal development among stigmatized youth. Such work should consider capturing serum levels of pubertal hormones, particularly estradiol, as there is some evidence that salivary levels of estradiol may have comparatively lower validity for measuring pubertal development relative to salivary levels of other hormonal indicators^118,119^. Notably, however, the consistency of our results across salivary levels of three pubertal hormones diminishes concerns about measurement bias introduced due to the potentially lower validity of salivary estradiol. Moreover, as the present study focused on pubertal timing in late childhood and early adolescence, including among primarily pre-menarcheal girls, subsequent scholarship is needed to evaluate the generalizability of our findings into later adolescence, particularly among larger samples of post-menarcheal girls.

Fourth, many ABCD Study^®^ youth hold multiple stigmatized identities and are thus exposed to structural stigma at the intersection of race, ethnicity, and/or gender. To our knowledge, measures of intersectional stigma at the structural level do not yet exist^58^. Scholars have sought to surmount this measurement shortcoming by modeling interactions between two or more structural stigma measures^109,120^, but we were underpowered to do so. The consistent associations between structural racism and earlier pubertal timing observed among Black girls in our study—but neither among girls generally nor among Black boys—may indicate that structural racism overlaps and intersects with structural sexism to have a particularly profound impact on pubertal timing among Black girls. Measurement advances in quantifying intersectional forms of structural stigma, including structural gendered racism^121^, are needed to evaluate this possibility. A recent paper by Carter and Seaton provides an important roadmap for incorporating intersectionality into future puberty research, which can complement the development of such measures^47^.

## Conclusion

Research into correlates and predictors of advanced pubertal development, including earlier pubertal timing, has focused almost exclusively on aspects of individuals and of their proximal environments^37–39,42,44,81,82^. We provide some of the first empirical evidence that macro-social factors—measured here in the form of structural stigma at the state level—are also associated with earlier pubertal timing. Although significant associations between measures of structural racism and xenophobia and earlier pubertal timing were observed among stigmatized and non-stigmatized youth at baseline, they persisted 1 year later only among the stigmatized—and most consistently so for Black and Latinx girls, respectively. Our study underscores the need for future scholarship to evaluate whether earlier pubertal timing is implicated in the link between structural stigma and adverse psychosocial outcomes among these stigmatized groups^55,59^. Moreover, it provides a novel framework for broadening the lens of developmental research to consider features of macro-social contexts, including structural stigma, which may provide new insights into determinants of puberty and perhaps other developmental processes^122^.

## Methods

### Participants and procedures

Participant data from the ABCD Study^®^ were publicly available and acquired via a data use agreement (DUA19-1048) with the NIMH Data Archive (ABCD Data Release 3.0; https://abcdstudy.org). The present study drew data from baseline and Year 1 assessments conducted with ABCD Study^®^ participants enrolled at one of 21 study sites located in 17 US states. ABCD Study^®^ youth enrolled at a now defunct site were excluded^123^. At baseline, participants included 11,844 youth (predominantly ages 9–10; *M* = 9.9, *SD* = 0.62) and their caregivers. At Year 1, 11,225 participating youth (predominantly ages 10–11; *M* = 10.9, *SD* = 0.64) and their caregivers were retained. Our primary analytic samples included 5662 girls at baseline and 5352 girls at Year 1; 2507 Black youth at baseline and 2274 Black youth at Year 1; and 2,406 Latinx youth at baseline and 2222 Latinx youth at Year 1 (see Supplementary Table 33 for demographics and other study variables at baseline and Year 1). We use the terms “girls” and “boys” to refer to assumed gender based on caregiver reports of youth’s birth-assigned sex at baseline, as information on youth-reported gender and gender identity was not collected at this timepoint. Although some youth with a birth-assigned sex of female may not identify as girls, our study’s measure of structural sexism may have been relevant to them, as it included aggregated implicit and explicit social attitudes related to gender roles and may characterize state-level sociopolitical contexts where institutional policies and practices prevent youth from changing gender markers. Black and Latinx youth were identified via caregiver-reported race and ethnicity. As caregivers could report multiple racial and ethnic identities for youth, we included all youth with a racial identity of Black in primary analyses related to structural racism and all youth with an ethnic identity of Latinx in primary analyses related to structural xenophobia. Consequently, 212 youth were included in both groups at baseline, and 195 youth were included in both groups at Year 1. ABCD Study^®^ families with lower (vs. higher) mean caregiver educational attainment were less likely to be retained at Year 1; attrition did not otherwise vary as a function of youth’s age, birth-assigned sex, race, ethnicity, or structural stigma context. ABCD Study^®^ recruitment methods and procedures are detailed elsewhere and were approved by Institutional Review Boards at each of the 21 study sites^124–127^.

### Measures

#### Structural stigma

Structural sexism, racism, and xenophobia were quantified at the state level using established measures of these constructs^60^. Consistent with prior research^58,60,110^ and theory^49,50^ on structural stigma, these measures incorporated publicly available indicators of laws/policies, aggregated implicit and explicit social attitudes, and/or other societal-level conditions as proxies for states’ macro-social climates surrounding the three stigmatized groups included in our primary analyses: Black girls and boys, Latinx girls and boys, and girls generally. The exploratory factor analytic (EFA) methods for selecting and combining indicators for each measure are extensively described elsewhere^60^. Briefly, candidate indicators for each structural stigma measure with a factor loading ≥ 0.40 were retained and used to create model-generated factor scores for structural racism, xenophobia, and sexism in each US state (see Supplementary Table 34 for structural stigma factor scores). These structural stigma measures have been previously associated with adverse neurodevelopmental (e.g., smaller hippocampal volume) and psychosocial (e.g., elevated psychopathology, decreased psychological intervention efficacy) outcomes among stigmatized youth, including Black and Latinx youth as well as girls, providing evidence of their construct validity^55,59,60,128,129^. The use of EFA further supports these measures’ validity (i.e., because indicators loaded onto single latent constructs of structural racism, xenophobia, and sexism) and increases their reliability (i.e., because measurement error is reduced by tapping into shared variance among indicators). Indicators and data sources for these structural stigma measures are summarized below and provided in Supplementary Table 35.

*Structural sexism*. The structural sexism measure included 18 single-item or composite indicators. Six indicators reflected women’s social (e.g., percent of women in each state living in counties without an abortion provider), economic (e.g., women’s labor force participation), and political (e.g., women’s voter registration) autonomy at the state level^60^. These indicators have been utilized in previous studies to quantify structural sexism and were acquired from public sources such as the Bureau of Labor Statistics, Current Population Survey, Institute for Women’s Policy Research, and Guttmacher Institute^96,97^. Twelve additional indicators captured aggregated implicit (e.g., automatic associations of gender with science and careers) and explicit attitudes towards gender and women’s social status. Attitudinal indicators were obtained from Project Implicit (2003–2018) and the General Social Survey (1974–2014), pooled across available years, and aggregated to the state level.

*Structural racism*. The structural racism measure comprised 31 indicators broadly capturing aggregated explicit anti-Black racial prejudice. To create these indicators, individual responses to attitudinal items from Project Implicit (2002–2017), the General Social Survey (1973–2014), and the American National Election Survey (1992–2016) were pooled across time and aggregated to the state level. Items encompassed various dimensions of anti-Black racial prejudice, including support for policies limiting the rights and welfare of Black people and the endorsement of anti-Black racial stereotypes. While other indicators of structural racism were considered in the EFA, only attitudinal indicators loaded onto this single factor, which may reflect the fact that anti-Black racial prejudice is often highest in US states where the population of Black residents is too small to reliably quantify other societal conditions (e.g., voter disenfranchisement, residential segregation) that have also been used to measure structural racism at the state level^109,130^.

*Structural xenophobia*. The measure of structural xenophobia consisted of two single-item indicators and one composite indicator. The single-item indicators represented aggregated explicit social attitudes towards Latinx people and towards immigrants acquired from the American National Election Survey. Individual responses to these two attitudinal items were pooled across available years (1996–2016 and 2004–2016, respectively) and aggregated to the state level. The third item included in this measure was a composite index summing the presence of 10 state laws/policies protective or prohibitive of immigrants’ rights (e.g., permitting application for a driver’s license irrespective of immigration status)^131^. Although not necessarily specific to Latinx people, attitudinal and policy indicators of the macro-social climate surrounding immigrants were included in this measure because of the high salience of anti-immigration policies to Latinx people, the frequent conflation of Latinx ethnicity and immigration status in the US, and the concealability of immigration status—all of which put Latinx people at disproportionate risk of experiencing xenophobia and its consequences^132–135^. Indeed, exposure to higher (vs. lower) levels of structural xenophobia has been consistently associated with negative health outcomes among Latinx people irrespective of immigration status^136^, including adverse neurodevelopmental and psychosocial outcomes among Latinx youth^55,59,60^.

#### Hormonal indicators of pubertal development

Salivary levels of three hormones were used to assess pubertal development at baseline and Year 1: DHEA and testosterone among both girls and boys and estradiol among girls only. Salivary biomarkers were acquired from whole saliva collected via passive drool by trained research assistants. Additional information on the methods for collecting and assaying these salivary samples is provided elsewhere^15,127^. Salivary hormone levels were residualized on youth’s chronological age to index pubertal timing for analysis; higher (i.e., more positive) residuals represented earlier (vs. later) pubertal timing^7,8^.

#### Youth- and caregiver-reported external, physical markers of pubertal development

We also assessed youth and caregiver reports of external, physical markers of pubertal development at baseline and Year 1 using the Pubertal Development Scale (PDS)^137^. We did so because pubertal hormone levels capture interrelated yet distinct aspects of pubertal development from these external, physical indicators, with which they are only modestly correlated^6,15,74,75^. Five PDS items assessed puberty-related changes in height, body hair, facial hair, skin, and voice among boys. Four PDS items captured puberty-related changes in height, body hair, skin, and breast development among girls, and the fifth queried menarche status. Except for the latter item, which was rated 1 (*no*) or 4 (*yes*) for girls, PDS items were rated on a Likert-scale from 1 (*has not yet begun*) to 4 (*seems completed*). Internal consistency was adequate for youth report (girls: α = 0.61–0.70; boys: α = 0.51–0.61) and caregiver report (girls: α = 0.71–0.77; boys: α = 0.58–0.68). Items were averaged to compute mean youth- and caregiver-reported PDS scores, which were residualized on youth’s chronological age to quantify pubertal timing; higher (i.e., more positive) residuals represented earlier (vs. later) pubertal timing.

For one set of supplemental analyses with external, physical markers of pubertal development, we created categorical youth- and caregiver-reported PDS scores^138^. For boys, the sum of the body hair, facial hair, and voice items were first categorized as 3 (*pre-puberty*), 4–5 (*early-puberty*), 6–8 (*mid-puberty*), 9–11 (*late-puberty*), and 12 (*post-puberty*). For girls, menarche status and the sum of the body hair and breast development items were first categorized as 2 without menarche (*pre-puberty*), 3 without menarche (*early-puberty*), ≥ 3 without menarche (*mid-puberty*), ≤ 7 with menarche (*late-puberty*), and 8 with menarche (*post-puberty*). Because prior analyses of ABCD Study^®^ data have documented low endorsement of late-puberty and post-puberty PDS categories at baseline and Year 1^139^, we combined mid-, late-, and post-puberty status into a single category. For a separate set of supplemental analyses with external, physical markers of pubertal development, we derived youth- and caregiver-reported PDS scores specific to adrenarche and gonadarche. Following prior studies^15,75^, gonadal PDS scores were computed for girls by averaging the growth, breast development, and menarche items and for boys by averaging the voice and facial hair items. Adrenal PDS scores were calculated by averaging the body hair and skin items for both girls and boys. For both sets of these supplemental analyses, categorical and gonadal/adrenal PDS scores were residualized on chronological age to index pubertal timing.

#### Covariates

Drawing on prior research on both structural stigma^60^ and pubertal development^15^, we accounted for two individual covariates in all analyses. First, we controlled for family SES in the form of mean caregiver educational attainment, as reported on a range from 0 (*never attended school or only attended kindergarten*) to 21 (*doctoral degree*). Mean caregiver educational attainment was used as a proxy for family SES given substantial missingness in data on mean household income. Second, we also controlled for BMI—calculated as the ratio of youth’s weight to the square of their height (measured up to three times each to ensure consistency and then averaged)—given BMI’s robust association with pubertal timing^15,19,82–84,139^. Outliers for BMI were winsorized at ± 3-SD from the mean. We additionally controlled for race/ethnicity (i.e., Latinx, non-Latinx White, non-Latinx Black, non-Latinx Asian, and other racial/ethnic identities) in primary analyses with girls. We did not control for birth-assigned sex or age because analyses were stratified by birth-assigned sex, and outcomes were residualized on chronological age. Analyses of pubertal timing using hormonal indicators of pubertal development included an additional control for the time of salivary hormone collection in hours since midnight. Finally, all analyses controlled for state-level income inequality using the Gini coefficient, which quantifies income maldistribution on a scale from 0 (*perfect equality*) to 1 (*perfect inequality*) and was acquired for included US states from the American Community Survey^140^. Controlling for state-level inequality enabled us to examine whether observed associations between structural stigma and pubertal timing were robust to a broader feature of stigmatized youth’s macro-social context that might be expected to influence pubertal timing among youth generally.

### Statistical analysis

#### Preregistration

Study analyses were preregistered on OSF (https://osf.io/zm62s/). We note a few necessary deviations from our preregistration below.

#### Power analysis

Preregistered power analyses indicated that we were adequately powered (> 90%) to detect small effect sizes for all pubertal timing outcomes among stigmatized and non-stigmatized groups at baseline. We were similarly powered (> 90%) to detect small effect sizes for pubertal timing via hormonal indicators among these groups at Year 1. Although well powered (> 90%) to detect small effect sizes for pubertal timing via caregiver- and youth-reported external, physical markers of pubertal development among girls at Year 1, we only had adequate power (> 90%) to detect medium effect sizes for these outcomes among Black and Latinx boys and girls at Year 1. This somewhat lower available power was attributable to smaller samples of Black and Latinx youth and caregivers completing these assessments at Year 1; although this attrition was not associated with levels of structural stigma, findings from these analyses should be interpreted cautiously given this reduced statistical power.

#### Data transformation

We performed several preregistered data transformations prior to analysis. First, consistent with prior work and research recommendations^75,141–144^, salivary levels of pubertal hormones were log-transformed, and outliers were winsorized at ± 3-SD from the mean. Transformations were performed separately for girls and boys at baseline and Year 1 given documented sex differences in pubertal timing, and thus the need to stratify relevant analyses by birth-assigned sex^16,19,20,145^. Second, to model outcomes of pubertal timing, we regressed chronological age from log-transformed hormonal indicators and from caregiver- and youth-reported external, physical markers of pubertal development separately for girls and boys at baseline and Year 1^7,8^. Positive residuals indexed earlier pubertal timing, and negative residuals indexed later pubertal timing.

#### Primary analysis

We fit linear mixed-effects models for each pubertal timing outcome variable among each stigmatized group (i.e., Black girls and boys, Latinx girls and boys, girls) separately at baseline and at Year 1. Analysis for Black and Latinx youth were stratified by birth-assigned sex. For each model, the relevant structural stigma measure (i.e., structural racism for Black girls and boys, structural xenophobia for Latinx girls and boys, structural sexism for girls) and study covariates were specified as fixed effects. To account for the nested nature of the data, we included random intercepts for family and for the US state affiliated with each ABCD Study^®^ site. We originally planned to include a random intercept for ABCD Study^®^ site; however, we ultimately included a random intercept for US state because of the high overlap between sites and US states and because the latter provided an even more rigorous control for unmeasured confounding at the state level. Linear mixed-effects models were fit using the “glmmTMB” package in R given its ability to estimate model parameters reliably when random-effects variance is small^146^.

#### Negative control analysis

As a form of negative control analysis^76^, we reran our primary models among non-stigmatized comparison groups consisting of youth who did not hold the stigmatized identity corresponding to each structural stigma measure. Specifically, we performed negative control analyses at baseline and Year 1 for structural racism among non-Latinx White girls and boys, structural xenophobia among non-Latinx White girls and boys, and structural sexism among boys. This approach enabled us to evaluate whether any associations between structural stigma and pubertal timing observed among stigmatized groups in our study were also observed among their non-stigmatized comparators. Because several studies have documented significant associations between structural stigma and adverse health outcomes among both stigmatized and non-stigmatized populations, especially when attitudinal measures of structural stigma are used^104,114,147,148^, these negative control analyses elucidated when and how consistently significant associations between structural stigma and earlier pubertal timing emerged only among stigmatized (vs. non-stigmatized) youth. For example, documenting significant associations between structural stigma and pubertal timing among stigmatized (vs. non-stigmatized) youth at both baseline and Year 1 might suggest that these groups face more persistent risk for chronic adverse health outcomes linked to earlier pubertal timing^21,23,24,87,88,90^.

#### Supplemental analysis

We preregistered several supplemental analyses to assess the robustness of our findings. First, given the relatively low endorsement of latter pubertal stages previously documented among ABCD Study^®^ youth at baseline and Year 1^139^, we repeated our primary analysis using three categories of caregiver- and youth-reported PDS scores (residualized on chronological age to index pubertal timing): pre-puberty, early-puberty, and mid-to-post-puberty. Second, we also reran our primary analysis of external, physical markers of pubertal development using caregiver- and youth-reported PDS items specific to adrenarche and gonadarche (also residualized on chronological age to index pubertal timing) because they represent largely distinct pubertal processes with respect to time (i.e., adrenarche typically precedes gonadarche) and their hormonal correlates (i.e., androgens vs. gonadotropins)^6^. Third, in order to establish that any observed associations between structural stigma and hormonal indicators of pubertal timing were not simply driven by post-menarche changes in hormone levels among girls, we reran relevant models excluding post-menarcheal girls.

#### Data transparency and openness

Analyses were conducted in R (version 4.4.0)^149^. Analytic code is available on OSF (https://osf.io/zm62s/). Thresholds for statistical significance were set at *p* < 0.05. As missingness for study covariates was minimal and at random, we conducted complete case analysis. We preregistered supplemental analyses using multiple imputation to handle potential missingness in study covariates. However, we did not conduct these analyses because there was minimal missing data for study covariates and because cluster sizes for ABCD Study^®^ families were too small for multilevel imputation to produce reliable estimates^150^.

## Supplementary Information

Supplementary Information.

## Data Availability

The present study is a secondary analysis of data from the ABCD Study^®^—a publicly available dataset that we accessed through a data use agreement with the NIMH Data Archive (DUA19-1048). Full information on data collection can be found at https://abcdstudy.org/.
