# Supplementary material for: Associations between structural stigma and earlier pubertal timing persist for 1 year among Black girls and Latinx youth
Source: Sci Rep. 2025 May 21;15:17655. doi: 10.1038/s41598-025-00378-8 (PMC12095683; doi:10.1038/s41598-025-00378-8)
Supplement: Supplementary file 1 — Supplementary Information. [file 41598_2025_378_MOESM1_ESM.docx]

**Associations Between Structural Stigma and Earlier Pubertal Timing Persist for One Year Among Black Girls and Latinx Youth**

*Rachel M. Martino, BS^1^; *Nathan L. Hollinsaid, BS^1^; Natalie L. Colich, PhD^1^;
Katie A. McLaughlin, PhD^1,2^; and Mark L. Hatzenbuehler, PhD^1^

*These authors contributed equally to this manuscript and are thus listed as co-first authors.

^1^Department of Psychology, Harvard University

^2^The Ballmer Institute for Children’s Behavioral Health, University of Oregon

**Supplementary Materials**

**Table S1. Associations Between Structural Racism and Hormonal Indicators of Puberal Timing Among Black Girls Not Endorsing Menarche**

| **Associations Between Structural Racism and Hormonal Indicators of  Puberal Timing Among Black Girls Not Endorsing Menarche: Estradiol** | | | | | | | | | | | | |
| --- | --- | --- | --- | --- | --- | --- | --- | --- | --- | --- | --- | --- |
|  | **Baseline** | | | | | | **Year 1** | | | | | |
|  | **b** | **SE** | ***z*** | ***p*** | **β** | **95% CI** | **b** | **SE** | ***z*** | ***p*** | **β** | **95% CI** |
| Intercept | -1.582 | 0.687 | -2.305 | 0.021* | 0.002 | (-0.082, 0.086) | -0.825 | 0.734 | -1.124 | 0.261 | -0.004 | (-0.099, 0.091) |
| Structural racism | 0.102 | 0.038 | 2.697 | 0.007** | 0.121 | (0.033, 0.208) | 0.066 | 0.038 | 1.723 | 0.085 | 0.083 | (-0.011, 0.178) |
| BMI | 0.011 | 0.004 | 2.506 | 0.012* | 0.083 | (0.018, 0.148) | 0.009 | 0.005 | 1.743 | 0.081 | 0.071 | (-0.009, 0.151) |
| Caregiver education | 0.012 | 0.008 | 1.537 | 0.124 | 0.054 | (-0.015, 0.122) | -0.006 | 0.010 | -0.655 | 0.513 | -0.028 | (-0.111, 0.055) |
| State income inequality | 1.937 | 1.382 | 1.401 | 0.161 | 0.057 | (-0.023, 0.137) | 1.029 | 1.471 | 0.699 | 0.485 | 0.033 | (-0.059, 0.124) |
| Collection time | 0.023 | 0.007 | 3.558 | <0.001*** | 0.124 | (0.056, 0.192) | 0.019 | 0.008 | 2.539 | 0.011* | 0.105 | (0.024, 0.187) |
| **Associations Between Structural Racism and Hormonal Indicators of  Puberal Timing Among Black Girls Not Endorsing Menarche: DHEA** | | | | | | | | | | | | |
|  | **Baseline** | | | | | | **Year 1** | | | | | |
|  | **b** | **SE** | ***z*** | ***p*** | **β** | **95% CI** | **b** | **SE** | ***z*** | ***p*** | **β** | **95% CI** |
| Intercept | 0.090 | 0.665 | 0.136 | 0.892 | 0.001 | (-0.063, 0.065) | -0.576 | 0.803 | -0.717 | 0.473 | 0.002 | (-0.075, 0.079) |
| Structural racism | 0.117 | 0.033 | 3.559 | <0.001*** | 0.117 | (0.053, 0.182) | 0.129 | 0.040 | 3.245 | 0.001** | 0.128 | (0.051, 0.205) |
| BMI | 0.033 | 0.005 | 6.848 | <0.001*** | 0.216 | (0.154, 0.278) | 0.034 | 0.006 | 5.710 | <0.001*** | 0.224 | (0.147, 0.301) |
| Caregiver education | -0.001 | 0.009 | -0.110 | 0.913 | -0.004 | (-0.069, 0.061) | 0.003 | 0.011 | 0.283 | 0.777 | 0.011 | (-0.067, 0.09) |
| State income inequality | -0.726 | 1.339 | -0.542 | 0.588 | -0.018 | (-0.082, 0.047) | 0.071 | 1.602 | 0.044 | 0.965 | 0.002 | (-0.077, 0.08) |
| Collection time | -0.016 | 0.007 | -2.211 | 0.027* | -0.070 | (-0.131, -0.008) | -0.008 | 0.009 | -0.825 | 0.409 | -0.032 | (-0.109, 0.044) |
| **Associations Between Structural Racism and Hormonal Indicators  of Puberal Timing Among Black Girls Not Endorsing Menarche: Testosterone** | | | | | | | | | | | | |
|  | **Baseline** | | | | | | **Year 1** | | | | | |
|  | **b** | **SE** | ***z*** | ***p*** | **β** | **95% CI** | **b** | **SE** | ***z*** | ***p*** | **β** | **95% CI** |
| Intercept | 0.531 | 0.544 | 0.975 | 0.329 | -0.004 | (-0.083, 0.076) | 0.308 | 0.592 | 0.520 | 0.603 | -0.004 | (-0.095, 0.087) |
| Structural racism | 0.104 | 0.027 | 3.837 | <0.001*** | 0.147 | (0.072, 0.222) | 0.093 | 0.030 | 3.043 | 0.002** | 0.140 | (0.05, 0.23) |
| BMI | 0.014 | 0.003 | 3.933 | <0.001*** | 0.127 | (0.064, 0.191) | 0.011 | 0.004 | 2.805 | 0.005** | 0.112 | (0.034, 0.19) |
| Caregiver education | -0.009 | 0.007 | -1.375 | 0.169 | -0.048 | (-0.115, 0.02) | -0.010 | 0.008 | -1.273 | 0.203 | -0.052 | (-0.132, 0.028) |
| State income inequality | -0.773 | 1.087 | -0.711 | 0.477 | -0.027 | (-0.101, 0.047) | -0.420 | 1.184 | -0.355 | 0.723 | -0.016 | (-0.105, 0.073) |
| Collection time | -0.012 | 0.006 | -2.164 | 0.03* | -0.076 | (-0.145, -0.007) | -0.008 | 0.006 | -1.278 | 0.201 | -0.053 | (-0.135, 0.028) |

**p*<0.05, ***p*<0.01, ****p*<0.001.

***Note*:** Hormone levels of estradiol, DHEA, and testosterone were log-transformed and age-residualized to index earlier (vs. later) pubertal timing. BMI = body-mass index.

**Table S2. Associations Between Structural Racism and Hormonal Indicators of Puberal Timing Among White Girls**

| **Associations Between Structural Racism and Hormonal Indicators of  Puberal Timing Among White Girls: Estradiol** | | | | | | | | | | | | |
| --- | --- | --- | --- | --- | --- | --- | --- | --- | --- | --- | --- | --- |
|  | **Baseline** | | | | | | **Year 1** | | | | | |
|  | **b** | **SE** | ***z*** | ***p*** | **β** | **95% CI** | **b** | **SE** | ***z*** | ***p*** | **β** | **95% CI** |
| Intercept | -0.980 | 0.653 | -1.502 | 0.133 | -0.024 | (-0.126, 0.079) | 0.188 | 0.593 | 0.318 | 0.751 | -0.006 | (-0.09, 0.079) |
| Structural racism | 0.061 | 0.035 | 1.733 | 0.083 | 0.091 | (-0.012, 0.194) | 0.003 | 0.032 | 0.099 | 0.921 | 0.004 | (-0.082, 0.091) |
| BMI | 0.013 | 0.003 | 3.947 | <0.001*** | 0.079 | (0.04, 0.118) | 0.014 | 0.004 | 3.814 | <0.001*** | 0.082 | (0.04, 0.124) |
| Caregiver education | 0.020 | 0.006 | 3.281 | 0.001** | 0.067 | (0.027, 0.107) | 0.013 | 0.007 | 1.755 | 0.079 | 0.038 | (-0.004, 0.081) |
| State income inequality | 0.299 | 1.366 | 0.219 | 0.827 | 0.012 | (-0.095, 0.119) | -2.149 | 1.227 | -1.751 | 0.08 | -0.079 | (-0.168, 0.009) |
| Collection time | 0.020 | 0.004 | 4.923 | <0.001*** | 0.103 | (0.062, 0.144) | 0.020 | 0.004 | 4.626 | <0.001*** | 0.105 | (0.06, 0.149) |
| **Associations Between Structural Racism and Hormonal Indicators of  Puberal Timing Among White Girls: DHEA** | | | | | | | | | | | | |
|  | **Baseline** | | | | | | **Year 1** | | | | | |
|  | **b** | **SE** | ***z*** | ***p*** | **β** | **95% CI** | **b** | **SE** | ***z*** | ***p*** | **β** | **95% CI** |
| Intercept | -0.292 | 0.531 | -0.550 | 0.582 | -0.003 | (-0.062, 0.056) | 0.673 | 0.595 | 1.130 | 0.259 | 0.002 | (-0.068, 0.072) |
| Structural racism | 0.060 | 0.028 | 2.145 | 0.032* | 0.066 | (0.006, 0.126) | 0.011 | 0.031 | 0.357 | 0.721 | 0.013 | (-0.059, 0.085) |
| BMI | 0.041 | 0.004 | 9.086 | <0.001*** | 0.179 | (0.14, 0.217) | 0.035 | 0.004 | 8.288 | <0.001*** | 0.171 | (0.131, 0.212) |
| Caregiver education | 0.016 | 0.008 | 2.030 | 0.042* | 0.041 | (0.001, 0.08) | 0.002 | 0.008 | 0.230 | 0.818 | 0.005 | (-0.037, 0.046) |
| State income inequality | -1.493 | 1.082 | -1.380 | 0.167 | -0.044 | (-0.106, 0.018) | -2.915 | 1.224 | -2.382 | 0.017* | -0.089 | (-0.163, -0.016) |
| Collection time | -0.008 | 0.005 | -1.569 | 0.117 | -0.032 | (-0.072, 0.008) | -0.006 | 0.005 | -1.194 | 0.232 | -0.026 | (-0.069, 0.017) |
| **Associations Between Structural Racism and Hormonal Indicators of  Puberal Timing Among White Girls: Testosterone** | | | | | | | | | | | | |
|  | **Baseline** | | | | | | **Year 1** | | | | | |
|  | **b** | **SE** | ***z*** | ***p*** | **β** | **95% CI** | **b** | **SE** | ***z*** | ***p*** | **β** | **95% CI** |
| Intercept | 0.067 | 0.479 | 0.140 | 0.889 | 0.005 | (-0.08, 0.089) | 0.154 | 0.260 | 0.591 | 0.554 | -0.002 | (-0.05, 0.046) |
| Structural racism | 0.064 | 0.026 | 2.493 | 0.013* | 0.108 | (0.023, 0.192) | 0.005 | 0.013 | 0.390 | 0.697 | 0.010 | (-0.039, 0.058) |
| BMI | 0.023 | 0.003 | 7.850 | <0.001*** | 0.153 | (0.115, 0.192) | 0.017 | 0.003 | 6.473 | <0.001*** | 0.135 | (0.094, 0.175) |
| Caregiver education | 0.010 | 0.005 | 1.981 | 0.048* | 0.039 | (0, 0.078) | -0.005 | 0.005 | -0.959 | 0.337 | -0.020 | (-0.062, 0.021) |
| State income inequality | -1.402 | 0.997 | -1.406 | 0.16 | -0.064 | (-0.152, 0.025) | -0.502 | 0.506 | -0.992 | 0.321 | -0.025 | (-0.076, 0.025) |
| Collection time | -0.002 | 0.003 | -0.676 | 0.499 | -0.014 | (-0.054, 0.026) | -0.014 | 0.003 | -4.670 | <0.001*** | -0.100 | (-0.142, -0.058) |

**p*<0.05, ***p*<0.01, ****p*<0.001.
***Note*:** Hormone levels of estradiol, DHEA, and testosterone were log-transformed and age-residualized to index earlier (vs. later) pubertal timing. BMI = body-mass index.

**Table S3. Associations Between Structural Racism and Hormonal Indicators of Puberal Timing Among Black Boys**

| **Associations Between Structural Racism and Hormonal Indicators of  Puberal Timing Among Black Boys: DHEA** | | | | | | | | | | | | |
| --- | --- | --- | --- | --- | --- | --- | --- | --- | --- | --- | --- | --- |
|  | **Baseline** | | | | | | **Year 1** | | | | | |
|  | **b** | **SE** | ***z*** | ***p*** | **β** | **95% CI** | **b** | **SE** | ***z*** | ***p*** | **β** | **95% CI** |
| Intercept | 0.084 | 0.842 | 0.100 | 0.920 | 0.012 | (-0.076, 0.1) | 0.086 | 0.903 | 0.095 | 0.924 | -0.002 | (-0.086, 0.082) |
| Structural racism | 0.083 | 0.047 | 1.784 | 0.074 | 0.078 | (-0.008, 0.164) | 0.048 | 0.048 | 1.000 | 0.317 | 0.041 | (-0.039, 0.121) |
| BMI | 0.038 | 0.005 | 7.890 | <0.001*** | 0.229 | (0.172, 0.286) | 0.040 | 0.006 | 6.894 | <0.001*** | 0.225 | (0.161, 0.289) |
| Caregiver education | 0.018 | 0.009 | 1.980 | 0.048* | 0.060 | (0.001, 0.12) | 0.022 | 0.011 | 1.992 | 0.046* | 0.068 | (0.001, 0.136) |
| State income inequality | -1.536 | 1.731 | -0.888 | 0.375 | -0.037 | (-0.119, 0.045) | -1.593 | 1.814 | -0.878 | 0.380 | -0.035 | (-0.114, 0.044) |
| Collection time | -0.014 | 0.008 | -1.761 | 0.078 | -0.059 | (-0.125, 0.007) | -0.027 | 0.009 | -2.988 | 0.003** | -0.105 | (-0.174, -0.036) |
| **Associations Between Structural Racism and Hormonal Indicators of  Puberal Timing Among Black Boys: Testosterone** | | | | | | | | | | | | |
|  | **Baseline** | | | | | | **Year 1** | | | | | |
|  | **b** | **SE** | ***z*** | ***p*** | **β** | **95% CI** | **b** | **SE** | ***z*** | ***p*** | **β** | **95% CI** |
| Intercept | 0.634 | 0.633 | 1.001 | 0.317 | -0.013 | (-0.111, 0.086) | 0.761 | 0.516 | 1.476 | 0.140 | -0.002 | (-0.078, 0.074) |
| Structural racism | 0.053 | 0.035 | 1.516 | 0.129 | 0.071 | (-0.021, 0.164) | 0.006 | 0.027 | 0.229 | 0.819 | 0.009 | (-0.065, 0.083) |
| BMI | 0.017 | 0.003 | 5.117 | <0.001*** | 0.150 | (0.092, 0.207) | 0.016 | 0.003 | 4.584 | <0.001*** | 0.149 | (0.085, 0.213) |
| Caregiver education | -0.003 | 0.006 | -0.479 | 0.632 | -0.015 | (-0.075, 0.045) | 0.006 | 0.007 | 0.933 | 0.351 | 0.032 | (-0.035, 0.098) |
| State income inequality | -1.269 | 1.304 | -0.973 | 0.331 | -0.044 | (-0.133, 0.045) | -1.425 | 1.037 | -1.374 | 0.169 | -0.052 | (-0.125, 0.022) |
| Collection time | -0.013 | 0.005 | -2.516 | 0.012* | -0.084 | (-0.149, -0.019) | -0.027 | 0.005 | -5.013 | <0.001*** | -0.174 | (-0.242, -0.106) |

**p*<0.05, ***p*<0.01, ****p*<0.001.

***Note*:** Hormone levels of DHEA and testosterone were log-transformed and age-residualized to index earlier (vs. later) pubertal timing. BMI = body-mass index.

**Table S4. Associations Between Structural Xenophobia and Hormonal Indicators of Puberal Timing Among Latinx Girls**

| **Associations Between Structural Xenophobia and Hormonal Indicators of  Puberal Timing Among Latinx Girls: Estradiol** | | | | | | | | | | | | |
| --- | --- | --- | --- | --- | --- | --- | --- | --- | --- | --- | --- | --- |
|  | **Baseline** | | | | | | **Year 1** | | | | | |
|  | **b** | **SE** | ***z*** | ***p*** | **β** | **95% CI** | **b** | **SE** | ***z*** | ***p*** | **β** | **95% CI** |
| Intercept | -0.657 | 0.866 | -0.759 | 0.448 | -0.017 | (-0.175, 0.142) | 0.793 | 1.064 | 0.745 | 0.456 | 0.022 | (-0.164, 0.208) |
| Structural xenophobia | 0.040 | 0.066 | 0.609 | 0.543 | 0.052 | (-0.116, 0.22) | -0.031 | 0.084 | -0.374 | 0.709 | -0.039 | (-0.246, 0.167) |
| BMI | 0.017 | 0.004 | 3.784 | <0.001*** | 0.119 | (0.057, 0.181) | 0.025 | 0.005 | 5.316 | <0.001*** | 0.181 | (0.114, 0.247) |
| Caregiver education | -0.003 | 0.005 | -0.633 | 0.527 | -0.021 | (-0.085, 0.043) | -0.008 | 0.006 | -1.342 | 0.18 | -0.048 | (-0.117, 0.022) |
| State income inequality | 0.284 | 1.855 | 0.153 | 0.878 | 0.010 | (-0.115, 0.134) | -2.812 | 2.282 | -1.232 | 0.218 | -0.094 | (-0.243, 0.055) |
| Collection time | 0.020 | 0.006 | 3.443 | <0.001*** | 0.110 | (0.047, 0.172) | 0.009 | 0.007 | 1.363 | 0.173 | 0.046 | (-0.02, 0.113 |
| **Associations Between Structural Xenophobia and Hormonal Indicators of  Puberal Timing Among Latinx Girls: DHEA** | | | | | | | | | | | | |
|  | **Baseline** | | | | | | **Year 1** | | | | | |
|  | **b** | **SE** | ***z*** | ***p*** | **β** | **95% CI** | **b** | **SE** | ***z*** | ***p*** | **β** | **95% CI** |
| Intercept | 0.029 | 0.755 | 0.038 | 0.970 | -0.005 | (-0.094, 0.084) | 0.345 | 1.099 | 0.314 | 0.753 | 0.001 | (-0.141, 0.144) |
| Structural xenophobia | 0.086 | 0.052 | 1.640 | 0.101 | 0.087 | (-0.017, 0.191) | -0.001 | 0.088 | -0.016 | 0.987 | -0.001 | (-0.174, 0.171) |
| BMI | 0.050 | 0.005 | 9.176 | <0.001*** | 0.276 | (0.217, 0.334) | 0.052 | 0.006 | 9.196 | <0.001*** | 0.297 | (0.234, 0.361) |
| Caregiver education | -0.013 | 0.006 | -2.037 | 0.042* | -0.063 | (-0.123, -0.002) | -0.011 | 0.007 | -1.623 | 0.105 | -0.054 | (-0.12, 0.011) |
| State income inequality | -1.315 | 1.617 | -0.814 | 0.416 | -0.035 | (-0.12, 0.05) | -2.368 | 2.369 | -1.000 | 0.317 | -0.062 | (-0.184, 0.06) |
| Collection time | -0.007 | 0.007 | -1.045 | 0.296 | -0.031 | (-0.09, 0.027) | -0.007 | 0.008 | -0.942 | 0.346 | -0.030 | (-0.094, 0.033) |
| **Associations Between Structural Xenophobia and Hormonal Indicators of  Puberal Timing Among Latinx Girls: Testosterone** | | | | | | | | | | | | |
|  | **Baseline** | | | | | | **Year 1** | | | | | |
|  | **b** | **SE** | ***z*** | ***p*** | **β** | **95% CI** | **b** | **SE** | ***z*** | ***p*** | **β** | **95% CI** |
| Intercept | 0.168 | 0.675 | 0.249 | 0.803 | -0.014 | (-0.146, 0.118) | 0.293 | 0.629 | 0.466 | 0.641 | 0.001 | (-0.121, 0.124) |
| Structural xenophobia | 0.092 | 0.051 | 1.808 | 0.071 | 0.132 | (-0.011, 0.276) | 0.038 | 0.049 | 0.773 | 0.439 | 0.060 | (-0.091, 0.21) |
| BMI | 0.031 | 0.004 | 8.253 | <0.001*** | 0.247 | (0.188, 0.305) | 0.028 | 0.004 | 8.010 | <0.001*** | 0.256 | (0.193, 0.318) |
| Caregiver education | -0.007 | 0.004 | -1.609 | 0.108 | -0.050 | (-0.11, 0.011) | -0.008 | 0.004 | -1.753 | 0.08 | -0.058 | (-0.123, 0.007) |
| State income inequality | -0.921 | 1.445 | -0.637 | 0.524 | -0.035 | (-0.143, 0.073) | -0.903 | 1.351 | -0.669 | 0.504 | -0.037 | (-0.146, 0.072) |
| Collection time | -0.015 | 0.005 | -3.015 | 0.003** | -0.091 | (-0.15, -0.032) | -0.022 | 0.005 | -4.408 | <0.001*** | -0.141 | (-0.204, -0.08) |

**p*<0.05, ***p*<0.01, ****p*<0.001.

***Note*:** Hormone levels of estradiol, DHEA, and testosterone were log-transformed and age-residualized to index earlier (vs. later) pubertal timing. BMI = body-mass index.

**Table S5. Associations Between Structural Xenophobia and Hormonal Indicators of Puberal Timing Among Latinx Boys**

| **Associations Between Structural Xenophobia and Hormonal Indicators of  Puberal Timing Among Latinx Boys: DHEA** | | | | | | | | | | | | |
| --- | --- | --- | --- | --- | --- | --- | --- | --- | --- | --- | --- | --- |
|  | **Baseline** | | | | | | **Year 1** | | | | | |
|  | **b** | **SE** | ***z*** | ***p*** | **β** | **95% CI** | **b** | **SE** | ***z*** | ***p*** | **β** | **95% CI** |
| Intercept | -0.075 | 1.051 | -0.071 | 0.943 | -0.029 | (-0.167, 0.109) | -0.067 | 0.748 | -0.090 | 0.928 | 0.003 | (-0.057, 0.064) |
| Structural xenophobia | -0.008 | 0.081 | -0.096 | 0.924 | -0.008 | (-0.161, 0.146) | 0.018 | 0.040 | 0.440 | 0.660 | 0.017 | (-0.058, 0.092) |
| BMI | 0.041 | 0.005 | 8.008 | <0.001*** | 0.232 | (0.175, 0.288) | 0.045 | 0.005 | 8.214 | <0.001*** | 0.255 | (0.194, 0.316) |
| Caregiver education | 0.001 | 0.007 | 0.205 | 0.838 | 0.006 | (-0.053, 0.066) | 0.013 | 0.007 | 1.820 | 0.069 | 0.058 | (-0.004, 0.121) |
| State income inequality | -1.326 | 2.250 | -0.589 | 0.556 | -0.032 | (-0.14, 0.075) | -1.493 | 1.567 | -0.953 | 0.341 | -0.036 | (-0.11, 0.038) |
| Collection time | -0.007 | 0.007 | -1.005 | 0.315 | -0.030 | (-0.089, 0.029) | -0.020 | 0.008 | -2.608 | 0.009** | -0.081 | (-0.142, -0.02) |
| **Associations Between Structural Xenophobia and Hormonal Indicators of**  **Puberal Timing Among Latinx Boys: Testosterone** | | | | | | | | | | | | |
|  | **Baseline** | | | | | | **Year 1** | | | | | |
|  | **b** | **SE** | ***z*** | ***p*** | **β** | **95% CI** | **b** | **SE** | ***z*** | ***p*** | **β** | **95% CI** |
| Intercept | 0.236 | 0.891 | 0.265 | 0.791 | -0.031 | (-0.208, 0.145) | 0.151 | 0.639 | 0.237 | 0.813 | 0.008 | (-0.1, 0.115) |
| Structural xenophobia | 0.063 | 0.069 | 0.911 | 0.362 | 0.087 | (-0.1, 0.273) | 0.012 | 0.048 | 0.241 | 0.810 | 0.016 | (-0.117, 0.15) |
| BMI | 0.026 | 0.004 | 7.172 | <0.001*** | 0.204 | (0.148, 0.259) | 0.026 | 0.004 | 7.065 | <0.001*** | 0.218 | (0.158, 0.279) |
| Caregiver education | -0.001 | 0.005 | -0.214 | 0.830 | -0.006 | (-0.066, 0.053) | 0.007 | 0.005 | 1.410 | 0.159 | 0.045 | (-0.018, 0.108) |
| State income inequality | -0.918 | 1.910 | -0.481 | 0.631 | -0.032 | (-0.161, 0.098) | -0.826 | 1.364 | -0.606 | 0.545 | -0.030 | (-0.125, 0.066) |
| Collection time | -0.019 | 0.005 | -3.632 | <0.001*** | -0.108 | (-0.167, -0.05) | -0.026 | 0.005 | -4.935 | <0.001*** | -0.153 | (-0.214, -0.09) |

**p*<0.05, ***p*<0.01, ****p*<0.001.
***Note*:** Hormone levels of DHEA and testosterone were log-transformed and age-residualized to index earlier (vs. later) pubertal timing. BMI = body-mass index.

**Table S6. Associations Between Structural Sexism and Hormonal Indicators of Puberal Timing Among Girls**

| **Associations Between Structural Sexism and Hormonal Indicators of**  **Puberal Timing Among Girls: Estradiol** | | | | | | | | | | | | |
| --- | --- | --- | --- | --- | --- | --- | --- | --- | --- | --- | --- | --- |
|  | **Baseline** | | | | | | **Year 1** | | | | | |
|  | **b** | **SE** | ***z*** | ***p*** | **β** | **95% CI** | **b** | **SE** | ***z*** | ***p*** | **β** | **95% CI** |
| Intercept | -1.120 | 0.581 | -1.928 | 0.054 | -0.018 | (-0.113, 0.077) | 0.150 | 0.529 | 0.283 | 0.777 | -0.065 | (-0.149, 0.019) |
| Structural sexism | 0.054 | 0.028 | 1.908 | 0.056 | 0.088 | (-0.002, 0.178) | 0.011 | 0.025 | 0.418 | 0.676 | 0.017 | (-0.063, 0.097) |
| BMI | 0.014 | 0.002 | 6.544 | <0.001*** | 0.097 | (0.068, 0.126) | 0.017 | 0.002 | 7.117 | <0.001*** | 0.115 | (0.083, 0.147) |
| Race/ethnicity (Asian) | 0.104 | 0.052 | 1.982 | 0.047* | 0.186 | (0.002, 0.37) | 0.193 | 0.059 | 3.255 | <0.001*** | 0.331 | (0.132, 0.53) |
| Race/ethnicity (Black) | 0.031 | 0.026 | 1.190 | 0.234 | 0.056 | (-0.036, 0.147) | 0.111 | 0.031 | 3.603 | <0.001*** | 0.190 | (0.087, 0.293) |
| Race/ethnicity (Latinx) | -0.006 | 0.025 | -0.255 | 0.799 | -0.011 | (-0.098, 0.075) | -0.005 | 0.028 | -0.178 | 0.859 | -0.009 | (-0.103, 0.086) |
| Race/ethnicity (Other) | 0.000 | 0.027 | 0.002 | 0.999 | 0.000 | (-0.094, 0.095) | 0.070 | 0.031 | 2.239 | 0.025* | 0.120 | (0.015, 0.225) |
| Caregiver education | 0.006 | 0.003 | 1.797 | 0.072 | 0.029 | (-0.003, 0.061) | 0.001 | 0.004 | 0.221 | 0.825 | 0.004 | (-0.031, 0.039) |
| State income inequality | 1.090 | 1.231 | 0.886 | 0.376 | 0.042 | (-0.05, 0.134) | -1.659 | 1.116 | -1.487 | 0.137 | -0.061 | (-0.142, 0.019) |
| Collection time | 0.019 | 0.003 | 6.846 | <0.001*** | 0.104 | (0.074, 0.134) | 0.018 | 0.003 | 5.742 | <0.001*** | 0.094 | (0.062, 0.126) |
| **Associations Between Structural Sexism and Hormonal Indicators of**  **Puberal Timing Among Girls: DHEA** | | | | | | | | | | | | |
|  | **Baseline** | | | | | | **Year 1** | | | | | |
|  | **b** | **SE** | ***z*** | ***p*** | **β** | **95% CI** | **b** | **SE** | ***z*** | ***p*** | **β** | **95% CI** |
| Intercept | -0.362 | 0.524 | -0.690 | 0.490 | -0.073 | (-0.141, -0.006) | 0.432 | 0.600 | 0.720 | 0.472 | -0.049 | (-0.125, 0.027) |
| Structural sexism | 0.036 | 0.025 | 1.442 | 0.149 | 0.045 | (-0.016, 0.105) | 0.010 | 0.029 | 0.367 | 0.714 | 0.013 | (-0.058, 0.085) |
| BMI | 0.040 | 0.003 | 14.528 | <0.001*** | 0.207 | (0.179, 0.235) | 0.039 | 0.003 | 14.009 | <0.001*** | 0.216 | (0.186, 0.246) |
| Race/ethnicity (Asian) | 0.246 | 0.067 | 3.698 | <0.001*** | 0.334 | (0.157, 0.512) | 0.227 | 0.070 | 3.214 | <0.001*** | 0.310 | (0.121, 0.499) |
| Race/ethnicity (Black) | 0.244 | 0.033 | 7.374 | <0.001*** | 0.332 | (0.243, 0.42) | 0.212 | 0.037 | 5.721 | <0.001*** | 0.290 | (0.191, 0.39) |
| Race/ethnicity (Latinx) | 0.084 | 0.031 | 2.700 | 0.007** | 0.115 | (0.031, 0.198) | 0.011 | 0.034 | 0.321 | 0.748 | 0.015 | (-0.075, 0.105) |
| Race/ethnicity (Other) | 0.039 | 0.035 | 1.125 | 0.261 | 0.053 | (-0.039, 0.145) | 0.012 | 0.037 | 0.322 | 0.747 | 0.016 | (-0.083, 0.116) |
| Caregiver education | -0.001 | 0.004 | -0.303 | 0.762 | -0.005 | (-0.036, 0.026) | -0.007 | 0.005 | -1.429 | 0.153 | -0.024 | (-0.058, 0.009) |
| State income inequality | -0.614 | 1.102 | -0.557 | 0.577 | -0.018 | (-0.08, 0.044) | -2.279 | 1.263 | -1.805 | 0.071 | -0.067 | (-0.14, 0.006) |
| Collection time | -0.009 | 0.004 | -2.605 | 0.009** | -0.038 | (-0.067, -0.009) | -0.004 | 0.004 | -1.074 | 0.283 | -0.017 | (-0.048, 0.014) |
| **Associations Between Structural Sexism and Hormonal Indicators of**  **Puberal Timing Among Girls: Testosterone** | | | | | | | | | | | | |
|  | **Baseline** | | | | | | **Year 1** | | | | | |
|  | **b** | **SE** | ***z*** | ***p*** | **β** | **95% CI** | **b** | **SE** | ***z*** | ***p*** | **β** | **95% CI** |
| Intercept | -0.029 | 0.474 | -0.060 | 0.952 | -0.043 | (-0.13, 0.044) | 0.040 | 0.279 | 0.143 | 0.886 | -0.051 | (-0.111, 0.009) |
| Structural sexism | 0.040 | 0.023 | 1.741 | 0.082 | 0.073 | (-0.009, 0.155) | 0.018 | 0.013 | 1.383 | 0.167 | 0.037 | (-0.015, 0.09) |
| BMI | 0.022 | 0.002 | 12.038 | <0.001*** | 0.171 | (0.143, 0.198) | 0.019 | 0.002 | 10.585 | <0.001*** | 0.164 | (0.134, 0.194) |
| Race/ethnicity (Asian) | 0.182 | 0.045 | 4.059 | <0.001*** | 0.366 | (0.189, 0.542) | 0.191 | 0.044 | 4.361 | <0.001*** | 0.419 | (0.231, 0.607) |
| Race/ethnicity (Black) | 0.152 | 0.023 | 6.722 | <0.001*** | 0.305 | (0.216, 0.394) | 0.141 | 0.023 | 6.048 | <0.001*** | 0.309 | (0.209, 0.409) |
| Race/ethnicity (Latinx) | -0.007 | 0.021 | -0.344 | 0.731 | -0.015 | (-0.097, 0.068) | -0.018 | 0.021 | -0.852 | 0.394 | -0.039 | (-0.129, 0.051) |
| Race/ethnicity (Other) | 0.033 | 0.023 | 1.426 | 0.154 | 0.067 | (-0.025, 0.158) | 0.044 | 0.023 | 1.904 | 0.057 | 0.097 | (-0.003, 0.198) |
| Caregiver education | -0.002 | 0.003 | -0.596 | 0.551 | -0.009 | (-0.04, 0.022) | -0.006 | 0.003 | -2.087 | 0.037* | -0.036 | (-0.069, -0.002) |
| State income inequality | -0.590 | 1.003 | -0.588 | 0.557 | -0.025 | (-0.109, 0.059) | -0.290 | 0.579 | -0.500 | 0.617 | -0.014 | (-0.068, 0.04) |
| Collection time | -0.007 | 0.002 | -3.014 | 0.003** | -0.044 | (-0.073, -0.015) | -0.013 | 0.002 | -5.798 | <0.001*** | -0.090 | (-0.121, -0.06) |

**p*<0.05, ***p*<0.01, ****p*<0.001.
***Note*:** Hormone levels of estradiol, DHEA, and testosterone were log-transformed and age-residualized to index earlier (vs. later) pubertal timing. BMI = body-mass index.

**Table S7. Associations Between Structural Xenophobia and Caregiver-Reported Categories of External, Physical Markers of Puberal Timing Among Latinx Girls**

| **Associations Between Structural Xenophobia and Caregiver-Reported Categories of  External, Physical Markers of Puberal Timing Among Latinx Girls** | | | | | | | | | | | | |
| --- | --- | --- | --- | --- | --- | --- | --- | --- | --- | --- | --- | --- |
|  | **Baseline** | | | | | | **Year 1** | | | | | |
|  | **b** | **SE** | ***z*** | ***p*** | **β** | **95% CI** | **b** | **SE** | ***z*** | ***p*** | **β** | **95% CI** |
| Intercept | -3.162 | 0.698 | -4.531 | <0.001*** | 0.008 | (-0.049, 0.066) | -2.108 | 0.793 | -2.657 | 0.008** | -0.001 | (-0.078, 0.075) |
| Structural xenophobia | 0.183 | 0.039 | 4.647 | <0.001*** | 0.166 | (0.096, 0.236) | 0.143 | 0.042 | 3.361 | <0.001*** | 0.158 | (0.066, 0.25) |
| BMI | 0.059 | 0.006 | 9.993 | <0.001*** | 0.295 | (0.237, 0.352) | 0.029 | 0.006 | 4.740 | <0.001*** | 0.184 | (0.108, 0.26) |
| Caregiver education | -0.002 | 0.007 | -0.348 | 0.728 | -0.011 | (-0.07, 0.049) | -0.003 | 0.008 | -0.383 | 0.702 | -0.015 | (-0.094, 0.063) |
| State income inequality | 4.734 | 1.465 | 3.230 | <0.001*** | 0.115 | (0.045, 0.184) | 3.666 | 1.649 | 2.223 | 0.026* | 0.105 | (0.012, 0.197) |

**p*<0.05, ***p*<0.01, ****p*<0.001.
***Note*:** Caregiver-reported external, physical markers on the Pubertal Development Scale were categorized and age-residualized to index earlier (vs. later) pubertal timing. BMI = body-mass index.

**Table S8. Associations Between Structural Xenophobia and Youth-Reported Categories of External, Physical Markers of Puberal Timing Among Latinx Girls**

| **Associations Between Structural Xenophobia and Youth-Reported Categories of External, Physical Markers of  Puberal Timing Among Latinx Girls** | | | | | | | | | | | | |
| --- | --- | --- | --- | --- | --- | --- | --- | --- | --- | --- | --- | --- |
|  | **Baseline** | | | | | | **Year 1** | | | | | |
|  | **b** | **SE** | ***z*** | ***p*** | **β** | **95% CI** | **b** | **SE** | ***z*** | ***p*** | **β** | **95% CI** |
| Intercept | -2.043 | 0.890 | -2.296 | 0.022* | 0.004 | (-0.066, 0.075) | -0.441 | 1.078 | -0.410 | 0.682 | 0.002 | (-0.099, 0.103) |
| Structural xenophobia | 0.119 | 0.049 | 2.449 | 0.014* | 0.107 | (0.021, 0.193) | 0.063 | 0.059 | 1.081 | 0.280 | 0.067 | (-0.054, 0.187) |
| BMI | 0.035 | 0.008 | 4.641 | <0.001*** | 0.170 | (0.098, 0.241) | 0.012 | 0.008 | 1.425 | 0.154 | 0.075 | (-0.028, 0.178) |
| Caregiver education | -0.002 | 0.009 | -0.181 | 0.856 | -0.007 | (-0.079, 0.066) | 0.006 | 0.012 | 0.462 | 0.644 | 0.025 | (-0.08, 0.13) |
| State income inequality | 3.181 | 1.852 | 1.718 | 0.086 | 0.075 | (-0.011, 0.16) | 0.461 | 2.250 | 0.205 | 0.838 | 0.013 | (-0.107, 0.132) |

**p*<0.05, ***p*<0.01, ****p*<0.001.
***Note*:** Youth-reported external, physical markers on the Pubertal Development Scale were categorized and age-residualized to index earlier (vs. later) pubertal timing. BMI = body-mass index.

**Table S9. Associations Between Structural Xenophobia and Caregiver-Reported Adrenal and Gonadal Markers of Puberal Timing Among Latinx Girls**

| **Associations Between Structural Xenophobia and Caregiver-Reported Adrenal Markers of  Puberal Timing Among Latinx Girls** | | | | | | | | | | | | |
| --- | --- | --- | --- | --- | --- | --- | --- | --- | --- | --- | --- | --- |
|  | **Baseline** | | | | | | **Year 1** | | | | | |
|  | **b** | **SE** | ***z*** | ***p*** | **β** | **95% CI** | **b** | **SE** | ***z*** | ***p*** | **β** | **95% CI** |
| Intercept | -4.017 | 0.618 | -6.496 | <0.001*** | 0.003 | (-0.054, 0.061) | -3.348 | 0.696 | -4.814 | <0.001*** | 0.000 | (-0.06, 0.06) |
| Structural xenophobia | 0.230 | 0.035 | 6.617 | <0.001*** | 0.237 | (0.167, 0.307) | 0.264 | 0.039 | 6.792 | <0.001*** | 0.254 | (0.181, 0.327) |
| BMI | 0.030 | 0.005 | 5.714 | <0.001*** | 0.168 | (0.11, 0.225) | 0.037 | 0.005 | 6.822 | <0.001*** | 0.209 | (0.149, 0.269) |
| Caregiver education | 0.015 | 0.006 | 2.510 | 0.012* | 0.076 | (0.017, 0.135) | 0.019 | 0.007 | 2.703 | 0.007** | 0.085 | (0.023, 0.147) |
| State income inequality | 7.084 | 1.298 | 5.456 | <0.001*** | 0.195 | (0.125, 0.265) | 5.322 | 1.461 | 3.644 | <0.001*** | 0.136 | (0.063, 0.209) |
| **Associations Between Structural Xenophobia and Caregiver-Reported Gonadal Markers of  Puberal Timing Among Latinx Girls** | | | | | | | | | | | | |
|  | **Baseline** | | | | | | **Year 1** | | | | | |
|  | **b** | **SE** | ***z*** | ***p*** | **β** | **95% CI** | **b** | **SE** | ***z*** | ***p*** | **β** | **95% CI** |
| Intercept | -1.436 | 0.425 | -3.378 | 0.001** | 0.004 | (-0.051, 0.06) | -2.165 | 0.537 | -4.029 | <0.001*** | 0.004 | (-0.054, 0.063) |
| Structural xenophobia | 0.072 | 0.024 | 3.002 | 0.003** | 0.104 | (0.036, 0.172) | 0.113 | 0.030 | 3.757 | <0.001*** | 0.137 | (0.066, 0.209) |
| BMI | 0.037 | 0.004 | 10.189 | <0.001*** | 0.293 | (0.236, 0.349) | 0.039 | 0.004 | 9.273 | <0.001*** | 0.281 | (0.222, 0.34) |
| Caregiver education | -0.009 | 0.004 | -2.050 | 0.040* | -0.060 | (-0.118, -0.003) | -0.008 | 0.005 | -1.535 | 0.125 | -0.047 | (-0.108, 0.013) |
| State income inequality | 1.986 | 0.893 | 2.224 | 0.026* | 0.077 | (0.009, 0.145) | 3.458 | 1.128 | 3.064 | 0.002** | 0.111 | (0.04, 0.183) |

**p*<0.05, ***p*<0.01, ****p*<0.001.
***Note*:** Caregiver-reported external, physical markers of adrenarche and gonadarche on the Pubertal Development Scale were averaged and age-residualized to index earlier (vs. later) pubertal timing. BMI = body-mass index.

**Table S10. Associations Between Structural Xenophobia and Youth-Reported Adrenal and Gonadal Markers of Puberal Timing Among Latinx Girls**

| **Associations Between Structural Xenophobia and Youth-Reported Adrenal Markers of  Puberal Timing Among Latinx Girls** | | | | | | | | | | | | |
| --- | --- | --- | --- | --- | --- | --- | --- | --- | --- | --- | --- | --- |
|  | **Baseline** | | | | | | **Year 1** | | | | | |
|  | **b** | **SE** | ***z*** | ***p*** | **β** | **95% CI** | **b** | **SE** | ***z*** | ***p*** | **β** | **95% CI** |
| Intercept | -2.996 | 0.660 | -4.541 | <0.001*** | 0.000 | (-0.057, 0.057) | -2.863 | 0.654 | -4.379 | <0.001*** | 0.002 | (-0.058, 0.062) |
| Structural xenophobia | 0.169 | 0.037 | 4.560 | <0.001*** | 0.163 | (0.093, 0.234) | 0.217 | 0.037 | 5.934 | <0.001*** | 0.223 | (0.15, 0.297) |
| BMI | 0.028 | 0.006 | 4.908 | <0.001*** | 0.147 | (0.088, 0.206) | 0.024 | 0.005 | 4.680 | <0.001*** | 0.146 | (0.085, 0.207) |
| Caregiver education | 0.002 | 0.007 | 0.256 | 0.798 | 0.008 | (-0.052, 0.067) | 0.012 | 0.006 | 1.933 | 0.053 | 0.061 | (-0.001, 0.124) |
| State income inequality | 5.410 | 1.383 | 3.912 | <0.001*** | 0.139 | (0.07, 0.209) | 4.961 | 1.373 | 3.614 | <0.001*** | 0.135 | (0.062, 0.208) |
| **Associations Between Structural Xenophobia and Youth-Reported Gonadal Markers of  Puberal Timing Among Latinx Girls** | | | | | | | | | | | | |
|  | **Baseline** | | | | | | **Year 1** | | | | | |
|  | **b** | **SE** | ***z*** | ***p*** | **β** | **95% CI** | **b** | **SE** | ***z*** | ***p*** | **β** | **95% CI** |
| Intercept | -0.886 | 0.571 | -1.552 | 0.121 | 0.001 | (-0.058, 0.06) | -1.563 | 0.645 | -2.423 | 0.015* | 0.005 | (-0.056, 0.065) |
| Structural xenophobia | 0.045 | 0.032 | 1.403 | 0.161 | 0.052 | (-0.02, 0.123) | 0.092 | 0.036 | 2.555 | 0.011* | 0.097 | (0.022, 0.171) |
| BMI | 0.024 | 0.005 | 4.981 | <0.001*** | 0.152 | (0.092, 0.212) | 0.032 | 0.005 | 6.307 | <0.001*** | 0.197 | (0.136, 0.259) |
| Caregiver education | 0.008 | 0.006 | 1.423 | 0.155 | 0.044 | (-0.017, 0.105) | -0.005 | 0.006 | -0.747 | 0.455 | -0.024 | (-0.086, 0.039) |
| State income inequality | 0.741 | 1.197 | 0.619 | 0.536 | 0.023 | (-0.049, 0.094) | 2.323 | 1.355 | 1.714 | 0.086 | 0.064 | (-0.009, 0.138) |

**p*<0.05, ***p*<0.01, ****p*<0.001.
***Note*:** Youth-reported external, physical markers of adrenarche and gonadarche on the Pubertal Development Scale were averaged and age-residualized to index earlier (vs. later) pubertal timing. BMI = body-mass index.

**Table S11. Associations Between Structural Xenophobia and Caregiver-Reported Categories of External, Physical Markers of Puberal Timing Among Latinx Boys**

| **Associations Between Structural Xenophobia and Caregiver-Reported Categories of External, Physical Markers of Puberal Timing Among Latinx Boys** | | | | | | | | | | | | |
| --- | --- | --- | --- | --- | --- | --- | --- | --- | --- | --- | --- | --- |
|  | **Baseline** | | | | | | **Year 1** | | | | | |
|  | **b** | **SE** | ***z*** | ***p*** | **β** | **95% CI** | **b** | **SE** | ***z*** | ***p*** | **β** | **95% CI** |
| Intercept | -1.255 | 0.614 | -2.045 | 0.041* | 0.004 | (-0.054, 0.062) | -1.511 | 0.828 | -1.824 | 0.068 | 0.011 | (-0.065, 0.086) |
| Structural xenophobia | 0.077 | 0.032 | 2.370 | 0.018* | 0.085 | (0.015, 0.155) | 0.066 | 0.045 | 1.454 | 0.146 | 0.069 | (-0.024, 0.162) |
| BMI | 0.018 | 0.005 | 4.004 | <0.001*** | 0.116 | (0.059, 0.173) | 0.016 | 0.006 | 2.592 | <0.01** | 0.099 | (0.024, 0.175) |
| Caregiver education | -0.008 | 0.006 | -1.375 | 0.169 | -0.042 | (-0.103, 0.018) | 0.013 | 0.009 | 1.439 | 0.15 | 0.058 | (-0.021, 0.137) |
| State income inequality | 2.441 | 1.279 | 1.908 | 0.056 | 0.069 | (-0.002, 0.139) | 2.423 | 1.742 | 1.391 | 0.164 | 0.065 | (-0.027, 0.157) |

**p*<0.05, ***p*<0.01, ****p*<0.001.
***Note*:** Caregiver-reported external, physical markers on the Pubertal Development Scale were categorized and age-residualized to index earlier (vs. later) pubertal timing. BMI = body-mass index.

**Table S12. Associations Between Structural Xenophobia and Youth-Reported Categories of External, Physical Markers of Puberal Timing Among Latinx Boys**

| **Associations Between Structural Xenophobia and Youth-Reported Categories of External, Physical Markers of  Puberal Timing Among Latinx Boys** | | | | | | | | | | | | |
| --- | --- | --- | --- | --- | --- | --- | --- | --- | --- | --- | --- | --- |
|  | **Baseline** | | | | | | **Year 1** | | | | | |
|  | **b** | **SE** | ***z*** | ***p*** | **β** | **95% CI** | **b** | **SE** | ***z*** | ***p*** | **β** | **95% CI** |
| Intercept | -0.711 | 1.087 | -0.654 | 0.513 | -0.007 | (-0.128, 0.113) | -2.588 | 1.064 | -2.433 | 0.015* | 0.003 | (-0.089, 0.095) |
| Structural xenophobia | 0.095 | 0.089 | 1.064 | 0.287 | 0.091 | (-0.076, 0.258) | 0.199 | 0.059 | 3.362 | <0.001** | 0.193 | (0.08, 0.305) |
| BMI | -0.001 | 0.005 | -0.242 | 0.809 | -0.007 | (-0.067, 0.052) | 0.003 | 0.008 | 0.365 | 0.715 | 0.017 | (-0.075, 0.11) |
| Caregiver education | -0.016 | 0.007 | -2.212 | 0.027* | -0.069 | (-0.131, -0.008) | -0.008 | 0.012 | -0.681 | 0.496 | -0.034 | (-0.131, 0.063) |
| State income inequality | 2.268 | 2.351 | 0.965 | 0.335 | 0.056 | (-0.058, 0.17) | 6.162 | 2.207 | 2.792 | 0.005** | 0.157 | (0.047, 0.268) |

**p*<0.05, ***p*<0.01, ****p*<0.001.
***Note*:** Youth-reported external, physical markers on the Pubertal Development Scale were categorized and age-residualized to index earlier (vs. later) pubertal timing. BMI = body-mass index.

**Table S13. Associations Between Structural Xenophobia and Caregiver-Reported Adrenal and Gonadal Markers of Puberal Timing Among Latinx Boys**

| **Associations Between Structural Xenophobia and Caregiver-Reported Adrenal Markers of  Puberal Timing Among Latinx Boys** | | | | | | | | | | | | |
| --- | --- | --- | --- | --- | --- | --- | --- | --- | --- | --- | --- | --- |
|  | **Baseline** | | | | | | **Year 1** | | | | | |
|  | **b** | **SE** | ***z*** | ***p*** | **β** | **95% CI** | **b** | **SE** | ***z*** | ***p*** | **β** | **95% CI** |
| Intercept | -1.425 | 0.542 | -2.630 | 0.009** | 0.003 | (-0.054, 0.06) | -1.793 | 0.590 | -3.038 | 0.002** | 0.003 | (-0.056, 0.061) |
| Structural xenophobia | 0.065 | 0.028 | 2.289 | 0.022* | 0.081 | (0.012, 0.15) | 0.058 | 0.031 | 1.856 | 0.063 | 0.068 | (-0.004, 0.14) |
| BMI | 0.018 | 0.004 | 4.581 | <0.001*** | 0.130 | (0.075, 0.186) | 0.024 | 0.004 | 5.706 | <0.001*** | 0.169 | (0.111, 0.227) |
| Caregiver education | 0.007 | 0.005 | 1.274 | 0.202 | 0.038 | (-0.021, 0.097) | 0.021 | 0.006 | 3.640 | <0.001*** | 0.113 | (0.052, 0.174) |
| State income inequality | 2.180 | 1.132 | 1.927 | 0.054 | 0.068 | (-0.001, 0.137) | 2.179 | 1.235 | 1.765 | 0.078 | 0.065 | (-0.007, 0.137) |
| **Associations Between Structural Xenophobia and Caregiver-Reported Gonadal Markers of  Puberal Timing Among Latinx Boys** | | | | | | | | | | | | |
|  | **Baseline** | | | | | | **Year 1** | | | | | |
|  | **b** | **SE** | ***z*** | ***p*** | **β** | **95% CI** | **b** | **SE** | ***z*** | ***p*** | **β** | **95% CI** |
| Intercept | -1.156 | 0.375 | -3.078 | 0.002** | 0.012 | (-0.044, 0.068) | -1.528 | 0.422 | -3.620 | <0.001*** | 0.002 | (-0.056, 0.061) |
| Structural xenophobia | 0.045 | 0.020 | 2.279 | 0.023* | 0.079 | (0.011, 0.146) | 0.053 | 0.022 | 2.384 | 0.017* | 0.087 | (0.016, 0.159) |
| BMI | 0.012 | 0.003 | 4.530 | <0.001*** | 0.125 | (0.071, 0.18) | 0.013 | 0.003 | 4.309 | <0.001*** | 0.127 | (0.069, 0.185) |
| Caregiver education | -0.017 | 0.004 | -4.822 | <0.001*** | -0.142 | (-0.2, -0.084) | -0.006 | 0.004 | -1.387 | 0.165 | -0.043 | (-0.104, 0.018) |
| State income inequality | 2.685 | 0.784 | 3.425 | <0.001*** | 0.118 | (0.051, 0.186) | 3.073 | 0.883 | 3.480 | <0.001*** | 0.128 | (0.056, 0.199) |

**p*<0.05, ***p*<0.01, ****p*<0.001.
***Note*:** Caregiver-reported external, physical markers of adrenarche and gonadarche on the Pubertal Development Scale were averaged and age-residualized to index earlier (vs. later) pubertal timing. BMI = body-mass index.

**Table S14. Associations Between Structural Xenophobia and Youth-Reported Adrenal and Gonadal Markers of Puberal Timing Among Latinx Boys**

| **Associations Between Structural Xenophobia and Youth-Reported Adrenal Markers of  Puberal Timing Among Latinx Boys** | | | | | | | | | | | | |
| --- | --- | --- | --- | --- | --- | --- | --- | --- | --- | --- | --- | --- |
|  | **Baseline** | | | | | | **Year 1** | | | | | |
|  | **b** | **SE** | ***z*** | ***p*** | **β** | **95% CI** | **b** | **SE** | ***z*** | ***p*** | **β** | **95% CI** |
| Intercept | -1.122 | 0.671 | -1.671 | 0.095 | 0.000 | (-0.056, 0.056) | -1.298 | 0.865 | -1.501 | 0.133 | -0.046 | (-0.168, 0.075) |
| Structural xenophobia | 0.092 | 0.036 | 2.555 | 0.011* | 0.089 | (0.021, 0.157) | 0.078 | 0.059 | 1.334 | 0.182 | 0.082 | (-0.038, 0.202) |
| BMI | 0.004 | 0.005 | 0.715 | 0.475 | 0.021 | (-0.036, 0.077) | 0.013 | 0.005 | 2.705 | 0.007** | 0.081 | (0.022, 0.14) |
| Caregiver education | -0.013 | 0.007 | -1.994 | 0.046* | -0.059 | (-0.117, -0.001) | 0.010 | 0.007 | 1.461 | 0.144 | 0.046 | (-0.016, 0.107) |
| State income inequality | 2.822 | 1.402 | 2.013 | 0.044* | 0.069 | (0.002, 0.136) | 2.083 | 1.853 | 1.124 | 0.261 | 0.055 | (-0.041, 0.151) |
| **Associations Between Structural Xenophobia and Youth-Reported Gonadal Markers of  Puberal Timing Among Latinx Boys** | | | | | | | | | | | | |
|  | **Baseline** | | | | | | **Year 1** | | | | | |
|  | **b** | **SE** | ***z*** | ***p*** | **β** | **95% CI** | **b** | **SE** | ***z*** | ***p*** | **β** | **95% CI** |
| Intercept | -0.226 | 0.715 | -0.316 | 0.752 | -0.022 | (-0.121, 0.077) | -1.639 | 0.559 | -2.932 | 0.003** | 0.000 | (-0.058, 0.058) |
| Structural xenophobia | -0.021 | 0.051 | -0.412 | 0.680 | -0.025 | (-0.143, 0.094) | 0.105 | 0.030 | 3.499 | <0.001*** | 0.128 | (0.056, 0.2) |
| BMI | 0.005 | 0.004 | 1.209 | 0.227 | 0.035 | (-0.022, 0.091) | 0.009 | 0.004 | 2.095 | 0.036* | 0.063 | (0.004, 0.121) |
| Caregiver education | -0.011 | 0.005 | -2.088 | 0.037* | -0.062 | (-0.12, -0.004) | -0.010 | 0.006 | -1.893 | 0.058 | -0.058 | (-0.118, 0.002) |
| State income inequality | 0.644 | 1.531 | 0.421 | 0.674 | 0.019 | (-0.07, 0.109) | 3.766 | 1.167 | 3.228 | <0.001*** | 0.116 | (0.045, 0.186) |

**p*<0.05, ***p*<0.01, ****p*<0.001.
***Note*:** Youth-reported external, physical markers of adrenarche and gonadarche on the Pubertal Development Scale were averaged and age-residualized to index earlier (vs. later) pubertal timing. BMI = body-mass index.

**Table S15. Associations Between Structural Xenophobia and Caregiver-Reported External, Physical Markers of Puberal Timing Among Non-Latinx White Girls**

| **Associations Between Structural Xenophobia and Caregiver-Reported External, Physical Markers of  Puberal Timing Among Non-Latinx White Girls** | | | | | | | | | | | | |
| --- | --- | --- | --- | --- | --- | --- | --- | --- | --- | --- | --- | --- |
|  | **Baseline** | | | | | | **Year 1** | | | | | |
|  | **b** | **SE** | ***z*** | ***p*** | **β** | **95% CI** | **b** | **SE** | ***z*** | ***p*** | **β** | **95% CI** |
| Intercept | -1.534 | 0.281 | -5.463 | <0.001*** | 0.009 | (-0.041, 0.059) | -1.941 | 0.392 | -4.955 | <0.001*** | 0.013 | (-0.044, 0.07) |
| Structural xenophobia | 0.025 | 0.021 | 1.192 | 0.233 | 0.033 | (-0.021, 0.086) | 0.045 | 0.029 | 1.568 | 0.117 | 0.048 | (-0.012, 0.108) |
| BMI | 0.048 | 0.002 | 20.448 | <0.001*** | 0.353 | (0.319, 0.387) | 0.057 | 0.003 | 20.60 | <0.001*** | 0.364 | (0.329, 0.399) |
| Caregiver education | -0.026 | 0.004 | -6.123 | <0.001*** | -0.111 | (-0.147, -0.076) | -0.024 | 0.005 | -4.333 | <0.001*** | -0.080 | (-0.116, -0.044) |
| State income inequality | 2.183 | 0.578 | 3.775 | <0.001*** | 0.107 | (0.051, 0.163) | 2.570 | 0.814 | 3.158 | 0.002** | 0.101 | (0.038, 0.164) |

**p*<0.05, ***p*<0.01, ****p*<0.001.
***Note*:** Caregiver-reported external, physical markers on the Pubertal Development Scale were averaged and age-residualized to index earlier (vs. later) pubertal timing. BMI = body-mass index.

**Table S16. Associations Between Structural Xenophobia and Caregiver-Reported External, Physical Markers of Puberal Timing Among Non-Latinx White Boys**

| **Associations Between Structural Xenophobia and Caregiver-Reported External, Physical Markers of  Puberal Timing Among Non-Latinx White Boys** | | | | | | | | | | | | |
| --- | --- | --- | --- | --- | --- | --- | --- | --- | --- | --- | --- | --- |
|  | **Baseline** | | | | | | **Year 1** | | | | | |
|  | **b** | **SE** | ***z*** | ***p*** | **β** | **95% CI** | **b** | **SE** | ***z*** | ***p*** | **β** | **95% CI** |
| Intercept | -0.711 | 0.194 | -3.673 | <0.001*** | 0.008 | (-0.042, 0.059) | -0.911 | 0.219 | -4.153 | <0.001*** | 0.004 | (-0.042, 0.051) |
| Structural xenophobia | 0.014 | 0.014 | 1.010 | 0.312 | 0.028 | (-0.027, 0.083) | -0.001 | 0.016 | -0.042 | 0.967 | -0.001 | (-0.051, 0.049) |
| BMI | 0.017 | 0.002 | 10.25 | <0.001*** | 0.176 | (0.142, 0.209) | 0.021 | 0.002 | 11.284 | <0.001*** | 0.196 | (0.162, 0.231) |
| Caregiver education | -0.016 | 0.003 | -5.563 | <0.001*** | -0.101 | (-0.137, -0.065) | -0.020 | 0.004 | -5.635 | <0.001*** | -0.103 | (-0.139, -0.067) |
| State income inequality | 1.289 | 0.400 | 3.223 | <0.001*** | 0.096 | (0.038, 0.154) | 1.681 | 0.446 | 3.768 | <0.001*** | 0.102 | (0.049, 0.155) |

**p*<0.05, ***p*<0.01, ****p*<0.001.
***Note*:** Caregiver-reported external, physical markers on the Pubertal Development Scale were averaged and age-residualized to index earlier (vs. later) pubertal timing. BMI = body-mass index.

**Table S17. Associations Between Structural Xenophobia and Youth-Reported External, Physical Markers of Puberal Timing Among Non-Latinx White Girls**

| **Associations Between Structural Xenophobia and Youth-Reported External, Physical Markers of  Puberal Timing Among Non-Latinx White Girls** | | | | | | | | | | | | |
| --- | --- | --- | --- | --- | --- | --- | --- | --- | --- | --- | --- | --- |
|  | **Baseline** | | | | | | **Year 1** | | | | | |
|  | **b** | **SE** | ***z*** | ***p*** | **β** | **95% CI** | **b** | **SE** | ***z*** | ***p*** | **β** | **95% CI** |
| Intercept | -0.955 | 0.230 | -4.153 | <0.001*** | 0.003 | (-0.034, 0.04) | -1.942 | 0.385 | -5.052 | <0.001*** | 0.017 | (-0.038, 0.072) |
| Structural xenophobia | 0.039 | 0.017 | 2.324 | 0.020* | 0.047 | (0.007, 0.087) | 0.045 | 0.028 | 1.590 | 0.112 | 0.047 | (-0.011, 0.106) |
| BMI | 0.030 | 0.003 | 10.76 | <0.001*** | 0.202 | (0.165, 0.239) | 0.050 | 0.003 | 17.09 | <0.001*** | 0.315 | (0.279, 0.351) |
| Caregiver education | -0.015 | 0.005 | -3.104 | 0.002** | -0.059 | (-0.096, -0.022) | -0.011 | 0.006 | -1.871 | 0.061 | -0.035 | (-0.072, 0.002) |
| State income inequality | 1.324 | 0.452 | 2.927 | 0.003** | 0.059 | (0.02, 0.099) | 2.487 | 0.796 | 3.124 | 0.002** | 0.097 | (0.036, 0.157) |

**p*<0.05, ***p*<0.01, ****p*<0.001.
***Note*:** Youth-reported external, physical markers on the Pubertal Development Scale were averaged and age-residualized to index earlier (vs. later) pubertal timing. BMI = body-mass index.

**Table S18. Associations Between Structural Xenophobia and Youth-Reported External, Physical Markers of Puberal Timing Among Non-Latinx White Boys**

| **Associations Between Structural Xenophobia and Youth-Reported External, Physical Markers of  Puberal Timing Among Non-Latinx White Boys** | | | | | | | | | | | | |
| --- | --- | --- | --- | --- | --- | --- | --- | --- | --- | --- | --- | --- |
|  | **Baseline** | | | | | | **Year 1** | | | | | |
|  | **b** | **SE** | ***z*** | ***p*** | **β** | **95% CI** | **b** | **SE** | ***z*** | ***p*** | **β** | **95% CI** |
| Intercept | -0.607 | 0.236 | -2.577 | <0.01** | 0.003 | (-0.035, 0.041) | -0.576 | 0.213 | -2.706 | 0.007** | 0.001 | (-0.037, 0.038) |
| Structural xenophobia | 0.019 | 0.016 | 1.158 | 0.247 | 0.024 | (-0.017, 0.065) | -0.006 | 0.015 | -0.424 | 0.671 | -0.009 | (-0.049, 0.031) |
| BMI | 0.010 | 0.003 | 3.612 | <0.001*** | 0.065 | (0.03, 0.1) | 0.018 | 0.002 | 7.611 | <0.001*** | 0.137 | (0.102, 0.173) |
| Caregiver education | -0.016 | 0.004 | -3.609 | <0.001*** | -0.065 | (-0.101, -0.03) | -0.020 | 0.004 | -4.507 | <0.001*** | -0.083 | (-0.118, -0.047) |
| State income inequality | 1.450 | 0.478 | 3.033 | 0.002** | 0.070 | (0.025, 0.115) | 1.082 | 0.420 | 2.577 | <0.01** | 0.054 | (0.013, 0.095) |

**p*<0.05, ***p*<0.01, ****p*<0.001.
***Note*:** Youth-reported external, physical markers on the Pubertal Development Scale were averaged and age-residualized to index earlier (vs. later) pubertal timing. BMI = body-mass index.

**Table S19. Associations Between Structural Xenophobia and Caregiver-Reported Categories of External, Physical Markers of Puberal Timing Among Non-Latinx White Girls**

| **Associations Between Structural Xenophobia and Caregiver-Reported Categories of External, Physical Markers of  Puberal Timing Among Non-Latinx White Girls** | | | | | | | | | | | | |
| --- | --- | --- | --- | --- | --- | --- | --- | --- | --- | --- | --- | --- |
|  | **Baseline** | | | | | | **Year 1** | | | | | |
|  | **b** | **SE** | ***z*** | ***p*** | **β** | **95% CI** | **b** | **SE** | ***z*** | ***p*** | **β** | **95% CI** |
| Intercept | -2.515 | 0.439 | -5.723 | <0.001*** | 0.008 | (-0.033, 0.049) | -2.338 | 0.485 | -4.825 | <0.001*** | 0.013 | (-0.036, 0.062) |
| Structural xenophobia | 0.052 | 0.032 | 1.648 | 0.099 | 0.037 | (-0.007, 0.082) | 0.038 | 0.034 | 1.126 | 0.26 | 0.030 | (-0.022, 0.082) |
| BMI | 0.095 | 0.004 | 21.192 | <0.001*** | 0.373 | (0.339, 0.408) | 0.067 | 0.005 | 14.810 | <0.001*** | 0.316 | (0.274, 0.358) |
| Caregiver education | -0.039 | 0.008 | -4.808 | <0.001*** | -0.088 | (-0.124, -0.052) | -0.030 | 0.009 | -3.319 | <0.001*** | -0.074 | (-0.118, -0.031) |
| State income inequality | 2.859 | 0.886 | 3.228 | <0.001*** | 0.075 | (0.03, 0.121) | 3.307 | 0.963 | 3.435 | <0.001*** | 0.092 | (0.039, 0.144) |

**p*<0.05, ***p*<0.01, ****p*<0.001.
***Note*:** Caregiver-reported external, physical markers on the Pubertal Development Scale were categorized and age-residualized to index earlier (vs. later) pubertal timing. BMI = body-mass index.

**Table S20. Associations Between Structural Xenophobia and Caregiver-Reported Categories of External, Physical Markers of Puberal Timing Among Non-Latinx White Boys**

| **Associations Between Structural Xenophobia and Caregiver-Reported Categories of External, Physical Markers of  Puberal Timing Among Non-Latinx White Boys** | | | | | | | | | | | | |
| --- | --- | --- | --- | --- | --- | --- | --- | --- | --- | --- | --- | --- |
|  | **Baseline** | | | | | | **Year 1** | | | | | |
|  | **b** | **SE** | ***z*** | ***p*** | **β** | **95% CI** | **b** | **SE** | ***z*** | ***p*** | **β** | **95% CI** |
| Intercept | -0.848 | 0.255 | -3.322 | <0.001*** | 0.008 | (-0.037, 0.052) | -1.262 | 0.401 | -3.145 | 0.002** | -0.001 | (-0.054, 0.052) |
| Structural xenophobia | 0.009 | 0.019 | 0.507 | 0.612 | 0.012 | (-0.036, 0.061) | 0.013 | 0.028 | 0.452 | 0.651 | 0.013 | (-0.043, 0.069) |
| BMI | 0.013 | 0.003 | 4.848 | <0.001*** | 0.086 | (0.051, 0.121) | 0.025 | 0.004 | 7.020 | <0.001*** | 0.147 | (0.106, 0.188) |
| Caregiver education | -0.019 | 0.004 | -4.336 | <0.001*** | -0.081 | (-0.117, -0.044) | -0.022 | 0.007 | -3.174 | 0.002** | -0.071 | (-0.115, -0.027) |
| State income inequality | 1.790 | 0.519 | 3.446 | <0.001*** | 0.088 | (0.038, 0.138) | 2.311 | 0.810 | 2.852 | 0.004** | 0.086 | (0.027, 0.145) |

**p*<0.05, ***p*<0.01, ****p*<0.001.
***Note*:** Caregiver-reported external, physical markers on the Pubertal Development Scale were categorized and age-residualized to index earlier (vs. later) pubertal timing. BMI = body-mass index.

**Table S21. Associations Between Structural Xenophobia and Youth-Reported Categories of External, Physical Markers of Puberal Timing Among Non-Latinx White Girls**

| **Associations Between Structural Xenophobia and Youth-Reported Categories of External, Physical Markers of  Puberal Timing Among Non-Latinx White Girls** | | | | | | | | | | | | |
| --- | --- | --- | --- | --- | --- | --- | --- | --- | --- | --- | --- | --- |
|  | **Baseline** | | | | | | **Year 1** | | | | | |
|  | **b** | **SE** | ***z*** | ***p*** | **β** | **95% CI** | **b** | **SE** | ***z*** | ***p*** | **β** | **95% CI** |
| Intercept | -1.265 | 0.444 | -2.848 | 0.004** | 0.002 | (-0.04, 0.044) | -2.018 | 0.602 | -3.353 | <0.001*** | 0.012 | (-0.05, 0.075) |
| Structural xenophobia | 0.093 | 0.033 | 2.864 | 0.004** | 0.067 | (0.021, 0.113) | -0.021 | 0.041 | -0.500 | 0.617 | -0.017 | (-0.082, 0.049) |
| BMI | 0.058 | 0.005 | 10.71 | <0.001*** | 0.230 | (0.188, 0.272) | 0.049 | 0.006 | 8.579 | <0.001*** | 0.238 | (0.184, 0.293) |
| Caregiver education | -0.024 | 0.010 | -2.456 | 0.014* | -0.053 | (-0.096, -0.011) | 0.014 | 0.011 | 1.253 | 0.210 | 0.035 | (-0.02, 0.091) |
| State income inequality | 1.162 | 0.882 | 1.318 | 0.187 | 0.031 | (-0.015, 0.076) | 1.734 | 1.208 | 1.436 | 0.151 | 0.048 | (-0.017, 0.113) |

**p*<0.05, ***p*<0.01, ****p*<0.001.
***Note*:** Youth-reported external, physical markers on the Pubertal Development Scale were categorized and age-residualized to index earlier (vs. later) pubertal timing. BMI = body-mass index.

**Table S22. Associations Between Structural Xenophobia and Youth-Reported Categories of External, Physical Markers of Puberal Timing Among Non-Latinx White Boys**

| **Associations Between Structural Xenophobia and Youth-Reported Categories of External, Physical Markers of  Puberal Timing Among Non-Latinx White Boys** | | | | | | | | | | | | |
| --- | --- | --- | --- | --- | --- | --- | --- | --- | --- | --- | --- | --- |
|  | **Baseline** | | | | | | **Year 1** | | | | | |
|  | **b** | **SE** | ***z*** | ***p*** | **β** | **95% CI** | **b** | **SE** | ***z*** | ***p*** | **β** | **95% CI** |
| Intercept | -0.955 | 0.369 | -2.585 | <0.001** | 0.002 | (-0.039, 0.044) | -0.594 | 0.458 | -1.298 | 0.194 | -0.003 | (-0.053, 0.047) |
| Structural xenophobia | 0.043 | 0.026 | 1.612 | 0.107 | 0.037 | (-0.008, 0.082) | 0.012 | 0.032 | 0.377 | 0.706 | 0.011 | (-0.044, 0.065) |
| BMI | 0.006 | 0.004 | 1.515 | 0.130 | 0.029 | (-0.008, 0.066) | 0.013 | 0.005 | 2.400 | 0.016* | 0.062 | (0.011, 0.112) |
| Caregiver education | -0.021 | 0.007 | -2.934 | 0.003** | -0.056 | (-0.094, -0.019) | -0.041 | 0.010 | -4.188 | <0.001*** | -0.109 | (-0.16, -0.058) |
| State income inequality | 2.460 | 0.754 | 3.261 | 0.001** | 0.080 | (0.032, 0.128) | 2.123 | 0.916 | 2.318 | 0.020* | 0.065 | (0.01, 0.119) |

**p*<0.05, ***p*<0.01, ****p*<0.001.
***Note*:** Youth-reported external, physical markers on the Pubertal Development Scale were categorized and age-residualized to index earlier (vs. later) pubertal timing. BMI = body-mass index.

**Table S23. Associations Between Structural Xenophobia and Caregiver-Reported Adrenal and Gonadal Markers of Puberal Timing Among Non-Latinx White Girls**

| **Associations Between Structural Xenophobia and Caregiver-Reported Adrenal Markers of  Puberal Timing Among Non-Latinx White Girls** | | | | | | | | | | | | |
| --- | --- | --- | --- | --- | --- | --- | --- | --- | --- | --- | --- | --- |
|  | **Baseline** | | | | | | **Year 1** | | | | | |
|  | **b** | **SE** | ***z*** | ***p*** | **β** | **95% CI** | **b** | **SE** | ***z*** | ***p*** | **β** | **95% CI** |
| Intercept | -1.709 | 0.330 | -5.176 | <0.001*** | 0.000 | (-0.04, 0.041) | -2.037 | 0.474 | -4.295 | <0.001*** | 0.007 | (-0.046, 0.059) |
| Structural xenophobia | 0.037 | 0.024 | 1.559 | 0.119 | 0.035 | (-0.009, 0.079) | 0.045 | 0.035 | 1.296 | 0.195 | 0.037 | (-0.019, 0.093) |
| BMI | 0.044 | 0.003 | 12.60 | <0.001*** | 0.229 | (0.193, 0.265) | 0.054 | 0.004 | 14.67 | <0.001*** | 0.269 | (0.233, 0.304) |
| Caregiver education | -0.030 | 0.006 | -4.815 | <0.001*** | -0.092 | (-0.129, -0.054) | -0.027 | 0.007 | -3.697 | <0.001*** | -0.071 | (-0.109, -0.033) |
| State income inequality | 2.844 | 0.661 | 4.304 | <0.001*** | 0.099 | (0.054, 0.144) | 3.023 | 0.977 | 3.094 | 0.002** | 0.092 | (0.034, 0.15) |
| **Associations Between Structural Xenophobia and Caregiver-Reported Gonadal Markers of  Puberal Timing Among Non-Latinx White Girls** | | | | | | | | | | | | |
|  | **Baseline** | | | | | | **Year 1** | | | | | |
|  | **b** | **SE** | ***z*** | ***p*** | **β** | **95% CI** | **b** | **SE** | ***z*** | ***p*** | **β** | **95% CI** |
| Intercept | -1.463 | 0.293 | -5.003 | <0.001*** | 0.013 | (-0.04, 0.066) | -1.910 | 0.399 | -4.781 | <0.001*** | 0.014 | (-0.043, 0.071) |
| Structural xenophobia | 0.016 | 0.022 | 0.736 | 0.462 | 0.021 | (-0.035, 0.078) | 0.042 | 0.029 | 1.424 | 0.154 | 0.044 | (-0.016, 0.104) |
| BMI | 0.051 | 0.002 | 21.98 | <0.001*** | 0.379 | (0.346, 0.413) | 0.058 | 0.003 | 20.69 | <0.001*** | 0.369 | (0.334, 0.404) |
| Caregiver education | -0.024 | 0.004 | -5.636 | <0.001*** | -0.101 | (-0.137, -0.066) | -0.021 | 0.006 | -3.855 | <0.001*** | -0.071 | (-0.107, -0.035) |
| State income inequality | 1.846 | 0.606 | 3.048 | 0.002** | 0.092 | (0.033, 0.15) | 2.343 | 0.830 | 2.821 | 0.005** | 0.091 | (0.028, 0.154) |

**p*<0.05, ***p*<0.01, ****p*<0.001.
***Note*:** Caregiver-reported external, physical markers of adrenarche and gonadarche on the Pubertal Development Scale were averaged and age-residualized to index earlier (vs. later) pubertal timing. BMI = body-mass index.

**Table S24. Associations Between Structural Xenophobia and Caregiver-Reported Adrenal and Gonadal Markers of Puberal Timing Among Non-Latinx White Boys**

| **Associations Between Structural Xenophobia and Caregiver-Reported Adrenal Markers of  Puberal Timing Among Non-Latinx White Boys** | | | | | | | | | | | | |
| --- | --- | --- | --- | --- | --- | --- | --- | --- | --- | --- | --- | --- |
|  | **Baseline** | | | | | | **Year 1** | | | | | |
|  | **b** | **SE** | ***z*** | ***p*** | **β** | **95% CI** | **b** | **SE** | ***z*** | ***p*** | **β** | **95% CI** |
| Intercept | -0.799 | 0.247 | -3.235 | <0.001*** | 0.003 | (-0.041, 0.048) | -0.842 | 0.325 | -2.593 | <0.01** | 0.000 | (-0.049, 0.048) |
| Structural xenophobia | -0.002 | 0.018 | -0.111 | 0.911 | -0.003 | (-0.051, 0.045) | -0.027 | 0.023 | -1.146 | 0.252 | -0.030 | (-0.082, 0.021) |
| BMI | 0.023 | 0.002 | 9.285 | <0.001*** | 0.163 | (0.128, 0.197) | 0.029 | 0.003 | 10.55 | <0.001*** | 0.185 | (0.151, 0.22) |
| Caregiver education | -0.015 | 0.004 | -3.488 | <0.001*** | -0.064 | (-0.099, -0.028) | -0.021 | 0.005 | -4.106 | <0.001*** | -0.076 | (-0.112, -0.04) |
| State income inequality | 1.156 | 0.504 | 2.293 | 0.022* | 0.059 | (0.009, 0.109) | 1.234 | 0.662 | 1.863 | 0.062 | 0.052 | (-0.003, 0.107) |
| **Associations Between Structural Xenophobia and Caregiver-Reported Gonadal Markers of  Puberal Timing Among Non-Latinx White Boys** | | | | | | | | | | | | |
|  | **Baseline** | | | | | | **Year 1** | | | | | |
|  | **b** | **SE** | ***z*** | ***p*** | **β** | **95% CI** | **b** | **SE** | ***z*** | ***p*** | **β** | **95% CI** |
| Intercept | -0.695 | 0.202 | -3.446 | <0.001*** | 0.007 | (-0.041, 0.055) | -0.960 | 0.207 | -4.636 | <0.001*** | 0.006 | (-0.038, 0.05) |
| Structural xenophobia | 0.027 | 0.015 | 1.834 | 0.067 | 0.049 | (-0.003, 0.101) | 0.016 | 0.015 | 1.113 | 0.266 | 0.027 | (-0.021, 0.075) |
| BMI | 0.013 | 0.002 | 7.334 | <0.001*** | 0.127 | (0.093, 0.161) | 0.016 | 0.002 | 8.800 | <0.001*** | 0.156 | (0.121, 0.191) |
| Caregiver education | -0.016 | 0.003 | -5.240 | <0.001*** | -0.096 | (-0.133, -0.06) | -0.020 | 0.004 | -5.474 | <0.001*** | -0.101 | (-0.138, -0.065) |
| State income inequality | 1.450 | 0.414 | 3.502 | <0.001*** | 0.099 | (0.044, 0.155) | 1.975 | 0.418 | 4.720 | <0.001*** | 0.121 | (0.071, 0.172) |

**p*<0.05, ***p*<0.01, ****p*<0.001.
***Note*:** Caregiver-reported external, physical markers of adrenarche and gonadarche on the Pubertal Development Scale were averaged and age-residualized to index earlier (vs. later) pubertal timing. BMI = body-mass index.

**Table S25. Associations Between Structural Xenophobia and Youth-Reported Adrenal and Gonadal Markers of Puberal Timing Among Non-Latinx White Girls**

| **Associations Between Structural Xenophobia and Youth-Reported Adrenal Markers of  Puberal Timing Among Non-Latinx White Girls** | | | | | | | | | | | | |
| --- | --- | --- | --- | --- | --- | --- | --- | --- | --- | --- | --- | --- |
|  | **Baseline** | | | | | | **Year 1** | | | | | |
|  | **b** | **SE** | ***z*** | ***p*** | **β** | **95% CI** | **b** | **SE** | ***z*** | ***p*** | **β** | **95% CI** |
| Intercept | -0.780 | 0.313 | -2.488 | 0.013* | -0.001 | (-0.038, 0.036) | -1.877 | 0.437 | -4.298 | <0.001*** | 0.013 | (-0.038, 0.063) |
| Structural xenophobia | 0.063 | 0.023 | 2.740 | 0.006** | 0.057 | (0.016, 0.097) | 0.056 | 0.032 | 1.744 | 0.081 | 0.048 | (-0.006, 0.102) |
| BMI | 0.027 | 0.004 | 7.120 | <0.001*** | 0.136 | (0.099, 0.174) | 0.042 | 0.004 | 11.596 | <0.001*** | 0.221 | (0.184, 0.258) |
| Caregiver education | -0.019 | 0.007 | -2.905 | 0.004** | -0.056 | (-0.094, -0.02) | -0.013 | 0.007 | -1.839 | 0.066 | -0.036 | (-0.074, 0.002) |
| State income inequality | 1.247 | 0.617 | 2.022 | 0.043* | 0.042 | (0.001, 0.082) | 2.790 | 0.894 | 3.120 | 0.002** | 0.089 | (0.033, 0.145) |
| **Associations Between Structural Xenophobia and Youth-Reported Gonadal Markers of  Puberal Timing Among Non-Latinx White Girls** | | | | | | | | | | | | |
|  | **Baseline** | | | | | | **Year 1** | | | | | |
|  | **b** | **SE** | ***z*** | ***p*** | **β** | **95% CI** | **b** | **SE** | ***z*** | ***p*** | **β** | **95% CI** |
| Intercept | -1.121 | 0.410 | -2.734 | 0.006** | 0.011 | (-0.048, 0.071) | -2.075 | 0.440 | -4.714 | <0.001*** | 0.015 | (-0.042, 0.072) |
| Structural xenophobia | 0.028 | 0.030 | 0.930 | 0.352 | 0.030 | (-0.033, 0.094) | 0.040 | 0.032 | 1.240 | 0.215 | 0.038 | (-0.022, 0.099) |
| BMI | 0.033 | 0.003 | 10.245 | <0.001*** | 0.195 | (0.158, 0.233) | 0.054 | 0.003 | 16.86 | <0.001*** | 0.312 | (0.276, 0.348) |
| Caregiver education | -0.005 | 0.006 | -0.896 | 0.37 | -0.017 | (-0.055, 0.021) | -0.010 | 0.006 | -1.587 | 0.113 | -0.030 | (-0.067, 0.007) |
| State income inequality | 1.178 | 0.852 | 1.383 | 0.167 | 0.046 | (-0.019, 0.112) | 2.514 | 0.915 | 2.749 | 0.006** | 0.089 | (0.025, 0.152) |

**p*<0.05, ***p*<0.01, ****p*<0.001.
***Note*:** Youth-reported external, physical markers of adrenarche and gonadarche on the Pubertal Development Scale were averaged and age-residualized to index earlier (vs. later) pubertal timing. BMI = body-mass index.

**Table S26. Associations Between Structural Xenophobia and Youth-Reported Adrenal and Gonadal Markers of Puberal Timing Among Non-Latinx White Boys**

| **Associations Between Structural Xenophobia and Youth-Reported Adrenal Markers of  Puberal Timing Among Non-Latinx White Boys** | | | | | | | | | | | | |
| --- | --- | --- | --- | --- | --- | --- | --- | --- | --- | --- | --- | --- |
|  | **Baseline** | | | | | | **Year 1** | | | | | |
|  | **b** | **SE** | ***z*** | ***p*** | **β** | **95% CI** | **b** | **SE** | ***z*** | ***p*** | **β** | **95% CI** |
| Intercept | -0.623 | 0.323 | -1.927 | 0.054 | 0.000 | (-0.037, 0.036) | -0.764 | 0.268 | -2.844 | 0.004** | -0.001 | (-0.036, 0.034) |
| Structural xenophobia | 0.021 | 0.022 | 0.953 | 0.34 | 0.019 | (-0.02, 0.058) | -0.014 | 0.020 | -0.706 | 0.48 | -0.014 | (-0.053, 0.025) |
| BMI | 0.003 | 0.004 | 0.884 | 0.377 | 0.016 | (-0.019, 0.051) | 0.022 | 0.003 | 6.929 | <0.001*** | 0.126 | (0.09, 0.162) |
| Caregiver education | -0.020 | 0.006 | -3.273 | <0.001*** | -0.059 | (-0.095, -0.024) | -0.022 | 0.006 | -3.822 | <0.001*** | -0.070 | (-0.106, -0.034) |
| State income inequality | 1.919 | 0.647 | 2.967 | 0.003** | 0.066 | (0.022, 0.109) | 1.439 | 0.531 | 2.709 | 0.007** | 0.054 | (0.015, 0.093) |
| **Associations Between Structural Xenophobia and Youth-Reported Gonadal Markers of  Puberal Timing Among Non-Latinx White Boys** | | | | | | | | | | | | |
|  | **Baseline** | | | | | | **Year 1** | | | | | |
|  | **b** | **SE** | ***z*** | ***p*** | **β** | **95% CI** | **b** | **SE** | ***z*** | ***p*** | **β** | **95% CI** |
| Intercept | -0.694 | 0.226 | -3.076 | 0.002** | 0.004 | (-0.031, 0.039) | -0.489 | 0.232 | -2.102 | 0.036* | 0.004 | (-0.036, 0.044) |
| Structural xenophobia | 0.011 | 0.017 | 0.661 | 0.508 | 0.013 | (-0.026, 0.052) | -0.004 | 0.017 | -0.256 | 0.798 | -0.006 | (-0.048, 0.037) |
| BMI | 0.016 | 0.003 | 5.401 | <0.001*** | 0.097 | (0.062, 0.132) | 0.015 | 0.002 | 6.092 | <0.001*** | 0.111 | (0.075, 0.146) |
| Caregiver education | -0.014 | 0.005 | -2.851 | 0.004** | -0.052 | (-0.088, -0.016) | -0.017 | 0.005 | -3.628 | <0.001*** | -0.067 | (-0.103, -0.031) |
| State income inequality | 1.292 | 0.447 | 2.891 | 0.004** | 0.057 | (0.018, 0.096) | 0.896 | 0.465 | 1.928 | 0.054 | 0.043 | (-0.001, 0.086) |

**p*<0.05, ***p*<0.01, ****p*<0.001.
***Note*:** Youth-reported external, physical markers of adrenarche and gonadarche on the Pubertal Development Scale were averaged and age-residualized to index earlier (vs. later) pubertal timing. BMI = body-mass index.

**Table S27. Associations Between Structural Racism and Caregiver-Reported External, Physical Markers of Puberal Timing Among Black Girls**

| **Associations Between Structural Racism and Caregiver-Reported External, Physical Markers of  Puberal Timing Among Black Girls** | | | | | | | | | | | | |
| --- | --- | --- | --- | --- | --- | --- | --- | --- | --- | --- | --- | --- |
|  | **Baseline** | | | | | | **Year 1** | | | | | |
|  | **b** | **SE** | ***z*** | ***p*** | **β** | **95% CI** | **b** | **SE** | ***z*** | ***p*** | **β** | **95% CI** |
| Intercept | -1.114 | 0.567 | -1.967 | 0.049* | 0.012 | (-0.052, 0.076) | -0.616 | 0.521 | -1.181 | 0.238 | 0.014 | (-0.044, 0.073) |
| Structural racism | -0.005 | 0.030 | -0.166 | 0.868 | -0.006 | (-0.078, 0.066) | -0.038 | 0.025 | -1.484 | 0.138 | -0.045 | (-0.104, 0.014) |
| BMI | 0.034 | 0.003 | 10.154 | <0.001*** | 0.280 | (0.226, 0.334) | 0.034 | 0.004 | 9.349 | <0.001*** | 0.273 | (0.216, 0.33) |
| Caregiver education | -0.018 | 0.007 | -2.658 | 0.008** | -0.080 | (-0.139, -0.021) | -0.021 | 0.007 | -2.978 | 0.003** | -0.090 | (-0.149, -0.031) |
| State income inequality | 2.147 | 1.129 | 1.900 | 0.057 | 0.064 | (-0.002, 0.13) | 1.132 | 1.057 | 1.071 | 0.284 | 0.032 | (-0.027, 0.091) |

**p*<0.05, ***p*<0.01, ****p*<0.001.
***Note*:** Caregiver-reported external, physical markers on the Pubertal Development Scale were averaged and age-residualized to index earlier (vs. later) pubertal timing. BMI = body-mass index.

**Table S28. Associations Between Structural Racism and Caregiver-Reported External, Physical Markers of Puberal Timing Among Black Boys**

| **Associations Between Structural Racism and Caregiver-Reported External, Physical Markers of  Puberal Timing Among Black Boys** | | | | | | | | | | | | |
| --- | --- | --- | --- | --- | --- | --- | --- | --- | --- | --- | --- | --- |
|  | **Baseline** | | | | | | **Year 1** | | | | | |
|  | **b** | **SE** | ***z*** | ***p*** | **β** | **95% CI** | **b** | **SE** | ***z*** | ***p*** | **β** | **95% CI** |
| Intercept | -0.247 | 0.639 | -0.387 | 0.699 | -0.011 | (-0.096, 0.075) | -0.346 | 0.479 | -0.724 | 0.469 | -0.004 | (-0.063, 0.056) |
| Structural racism | -0.002 | 0.034 | -0.066 | 0.948 | -0.003 | (-0.085, 0.08) | 0.036 | 0.024 | 1.481 | 0.139 | 0.045 | (-0.015, 0.105) |
| BMI | 0.013 | 0.004 | 3.631 | <0.001*** | 0.101 | (0.046, 0.155) | 0.019 | 0.004 | 5.396 | <0.001*** | 0.158 | (0.101, 0.216) |
| Caregiver education | -0.030 | 0.007 | -4.239 | <0.001*** | -0.130 | (-0.191, -0.07) | -0.015 | 0.007 | -2.209 | 0.027* | -0.068 | (-0.128, -0.008) |
| State income inequality | 1.405 | 1.297 | 1.084 | 0.278 | 0.045 | (-0.036, 0.126) | 0.896 | 0.964 | 0.929 | 0.353 | 0.029 | (-0.032, 0.089) |

**p*<0.05, ***p*<0.01, ****p*<0.001.
***Note*:** Caregiver-reported external, physical markers on the Pubertal Development Scale were averaged and age-residualized to index earlier (vs. later) pubertal timing. BMI = body-mass index.

**Table S29. Associations Between Structural Racism and Youth-Reported External, Physical Markers of Puberal Timing Among Black Girls**

| **Associations Between Structural Racism and Youth-Reported External, Physical Markers of  Puberal Timing Among Black Girls** | | | | | | | | | | | | |
| --- | --- | --- | --- | --- | --- | --- | --- | --- | --- | --- | --- | --- |
|  | **Baseline** | | | | | | **Year 1** | | | | | |
|  | **b** | **SE** | ***z*** | ***p*** | **β** | **95% CI** | **b** | **SE** | ***z*** | ***p*** | **β** | **95% CI** |
| Intercept | 0.187 | 0.551 | 0.339 | 0.734 | 0.007 | (-0.054, 0.067) | -1.160 | 0.677 | -1.713 | 0.087 | 0.011 | (-0.063, 0.086) |
| Structural racism | 0.004 | 0.030 | 0.144 | 0.886 | 0.005 | (-0.06, 0.07) | -0.020 | 0.034 | -0.593 | 0.554 | -0.022 | (-0.096, 0.052) |
| BMI | 0.020 | 0.004 | 5.218 | <0.001*** | 0.151 | (0.095, 0.208) | 0.026 | 0.004 | 6.308 | <0.001*** | 0.191 | (0.131, 0.25) |
| Caregiver education | -0.003 | 0.007 | -0.457 | 0.648 | -0.014 | (-0.072, 0.045) | 0.011 | 0.008 | 1.381 | 0.167 | 0.043 | (-0.018, 0.105) |
| State income inequality | -0.727 | 1.118 | -0.650 | 0.516 | -0.020 | (-0.08, 0.04) | 1.330 | 1.379 | 0.965 | 0.335 | 0.035 | (-0.036, 0.106) |

**p*<0.05, ***p*<0.01, ****p*<0.001.
***Note*:** Youth-reported external, physical markers on the Pubertal Development Scale were averaged and age-residualized to index earlier (vs. later) pubertal timing. BMI = body-mass index.

**Table S30. Associations Between Structural Racism and Youth-Reported External, Physical Markers of Puberal Timing Among Black Boys**

| **Associations Between Structural Racism and Youth-Reported External, Physical Markers of  Puberal Timing Among Black Boys** | | | | | | | | | | | | |
| --- | --- | --- | --- | --- | --- | --- | --- | --- | --- | --- | --- | --- |
|  | **Baseline** | | | | | | **Year 1** | | | | | |
|  | **b** | **SE** | ***z*** | ***p*** | **β** | **95% CI** | **b** | **SE** | ***z*** | ***p*** | **β** | **95% CI** |
| Intercept | -0.028 | 0.475 | -0.059 | 0.953 | -0.001 | (-0.057, 0.055) | -0.272 | 0.488 | -0.558 | 0.577 | 0.000 | (-0.058, 0.058) |
| Structural racism | 0.017 | 0.024 | 0.687 | 0.492 | 0.020 | (-0.037, 0.076) | 0.036 | 0.025 | 1.472 | 0.141 | 0.044 | (-0.015, 0.103) |
| BMI | 0.015 | 0.004 | 3.863 | <0.001*** | 0.110 | (0.054, 0.166) | 0.017 | 0.004 | 4.411 | <0.001*** | 0.131 | (0.073, 0.19) |
| Caregiver education | -0.003 | 0.007 | -0.484 | 0.628 | -0.014 | (-0.07, 0.042) | -0.006 | 0.007 | -0.849 | 0.396 | -0.025 | (-0.084, 0.033) |
| State income inequality | -0.213 | 0.962 | -0.221 | 0.825 | -0.006 | (-0.063, 0.05) | 0.379 | 0.982 | 0.386 | 0.7 | 0.012 | (-0.047, 0.07) |

**p*<0.05, ***p*<0.01, ****p*<0.001.
***Note*:** Youth-reported external, physical markers on the Pubertal Development Scale were averaged and age-residualized to index earlier (vs. later) pubertal timing. BMI = body-mass index.

**Table S31. Associations Between Structural Sexism and Caregiver-Reported External, Physical Markers of Puberal Timing Among Girls**

| **Associations Between Structural Sexism and Caregiver-Reported External, Physical Markers of  Puberal Timing Among Girls** | | | | | | | | | | | | |
| --- | --- | --- | --- | --- | --- | --- | --- | --- | --- | --- | --- | --- |
|  | **Baseline** | | | | | | **Year 1** | | | | | |
|  | **b** | **SE** | ***z*** | ***p*** | **β** | **95% CI** | **b** | **SE** | ***z*** | ***p*** | **β** | **95% CI** |
| Intercept | -1.465 | 0.303 | -4.836 | <0.001*** | -0.101 | (-0.158, -0.044) | -1.550 | 0.395 | -3.926 | <0.001*** | -0.061 | (-0.124, 0.003) |
| Structural sexism | 0.004 | 0.014 | 0.287 | 0.774 | 0.007 | (-0.043, 0.057) | 0.001 | 0.019 | 0.032 | 0.975 | 0.001 | (-0.056, 0.058) |
| BMI | 0.040 | 0.002 | 24.284 | <0.001*** | 0.303 | (0.278, 0.327) | 0.070 | 0.050 | 1.395 | 0.163 | 0.120 | (-0.049, 0.289) |
| Race/ethnicity (Asian) | 0.031 | 0.042 | 0.750 | 0.453 | 0.062 | (-0.1, 0.223) | 0.255 | 0.025 | 10.090 | <0.001*** | 0.435 | (0.351, 0.52) |
| Race/ethnicity (Black) | 0.317 | 0.020 | 15.604 | <0.001*** | 0.624 | (0.545, 0.702) | 0.041 | 0.023 | 1.748 | 0.08 | 0.070 | (-0.008, 0.148) |
| Race/ethnicity (Latinx) | 0.029 | 0.019 | 1.518 | 0.129 | 0.057 | (-0.017, 0.131) | 0.061 | 0.026 | 2.366 | 0.018* | 0.104 | (0.018, 0.19) |
| Race/ethnicity (Other) | 0.111 | 0.021 | 5.190 | <0.001*** | 0.218 | (0.135, 0.3) | 0.046 | 0.002 | 24.616 | <0.001*** | 0.324 | (0.299, 0.35) |
| Caregiver education | -0.014 | 0.003 | -5.502 | <0.001*** | -0.077 | (-0.105, -0.05) | -0.012 | 0.003 | -3.697 | <0.001*** | -0.054 | (-0.083, -0.026) |
| State income inequality | 1.898 | 0.637 | 2.978 | 0.003** | 0.078 | (0.027, 0.129) | 1.683 | 0.832 | 2.022 | 0.043* | 0.060 | (0.002, 0.118) |

**p*<0.05, ***p*<0.01, ****p*<0.001.
***Note*:** Caregiver-reported external, physical markers on the Pubertal Development Scale were averaged and age-residualized to index earlier (vs. later) pubertal timing. Non-Latinx White was the reference group for race/ethnicity. BMI = body-mass index.

**Table S32. Associations Between Structural Sexism and Youth-Reported External, Physical Markers of Puberal Timing Among Girls**

| **Associations Between Structural Sexism and Youth-Reported External, Physical Markers of  Puberal Timing Among Girls** | | | | | | | | | | | | |
| --- | --- | --- | --- | --- | --- | --- | --- | --- | --- | --- | --- | --- |
|  | **Baseline** | | | | | | **Year 1** | | | | | |
|  | **b** | **SE** | ***z*** | ***p*** | **β** | **95% CI** | **b** | **SE** | ***z*** | ***p*** | **β** | **95% CI** |
| Intercept | -0.703 | 0.280 | -2.508 | 0.012* | -0.078 | (-0.13, -0.027) | -1.432 | 0.380 | -3.763 | <0.001*** | -0.007 | (-0.069, 0.055) |
| Structural sexism | 0.018 | 0.013 | 1.395 | 0.163 | 0.031 | (-0.012, 0.074) | 0.012 | 0.018 | 0.690 | 0.49 | 0.019 | (-0.036, 0.074) |
| BMI | 0.025 | 0.002 | 13.181 | <0.001*** | 0.182 | (0.155, 0.209) | 0.039 | 0.002 | 19.277 | <0.001*** | 0.271 | (0.243, 0.298) |
| Race/ethnicity (Asian) | 0.021 | 0.048 | 0.442 | 0.659 | 0.040 | (-0.136, 0.215) | 0.050 | 0.053 | 0.951 | 0.342 | 0.086 | (-0.092, 0.264) |
| Race/ethnicity (Black) | 0.222 | 0.023 | 9.679 | <0.001*** | 0.414 | (0.33, 0.498) | 0.115 | 0.026 | 4.372 | <0.001*** | 0.197 | (0.109, 0.285) |
| Race/ethnicity (Latinx) | 0.041 | 0.022 | 1.880 | 0.06 | 0.076 | (-0.003, 0.155) | -0.001 | 0.024 | -0.032 | 0.974 | -0.001 | (-0.083, 0.081) |
| Race/ethnicity (Other) | 0.050 | 0.024 | 2.038 | 0.042* | 0.092 | (0.004, 0.181) | 0.017 | 0.027 | 0.635 | 0.526 | 0.029 | (-0.061, 0.12) |
| Caregiver education | -0.004 | 0.003 | -1.206 | 0.228 | -0.018 | (-0.048, 0.011) | -0.001 | 0.003 | -0.434 | 0.664 | -0.007 | (-0.037, 0.024) |
| State income inequality | 0.522 | 0.584 | 0.893 | 0.372 | 0.020 | (-0.024, 0.065) | 1.471 | 0.800 | 1.839 | 0.066 | 0.053 | (-0.003, 0.109) |

**p*<0.05, ***p*<0.01, ****p*<0.001.
***Note*:** Youth-reported external, physical markers on the Pubertal Development Scale were averaged and age-residualized to index earlier (vs. later) pubertal timing. Non-Latinx White was the reference group for race/ethnicity. BMI = body-mass index.

**Table S33. Demographic and Other Study Variables at Baseline and Year 1**

| **Demographic and Other Study Variables at Baseline and Year 1 for Black Girls** | | |
| --- | --- | --- |
| **Variable** | **Baseline: M(SD); range** | **Year 1: M(SD); range** |
| Age | 9.89 (0.61); 8.92—11.00 | 10.89 (0.63); 9.75—12.25 |
| Structural racism | 0.07 (0.67); -1.98—1.11 | 0.08 (0.68); -1.98—1.11 |
| Mean caregiver educational attainment | 15.13 (2.40); 6.00—21.00 | 15.19 (2.42); 6.00—21.00 |
| BMI | 20.64 (4.47); 14.57—29.01 | 21.69 (4.58); 14.57—29.01 |
| State income inequality | 0.47 (0.02); 0.43—0.51 | 0.47 (0.02); 0.43—0.51 |
| Estradiol (log-transformed and age-residualized) | 0.05 (0.56); -3.03—1.12 | 0.08 (0.54); -2.43—1.17 |
| DHEA (log-transformed and age-residualized) | 0.23 (0.67); -2.89—1.70 | 0.22 (0.69); -2.38—1.63 |
| Testosterone (log-transformed and age-residualized) | 0.23 (0.48); -2.32—1.20 | 0.14 (0.45); -1.56—1.34 |
| Youth-reported PDS (log-transformed and age-residualized) | 0.21 (0.60); -0.85—2.44 | 0.19 (0.62); -1.28—2.10 |
| Caregiver-reported PDS (log-transformed and age-residualized) | 0.33 (0.55); -1.00—2.40 | 0.32 (0.57); -1.39—2.06 |
| **Demographic and Other Study Variables at Baseline and Year 1 for Black Boys** | | |
| **Variable** | **Baseline: M(SD); range** | **Year 1: M(SD); range** |
| Age | 9.91 (0.61); 8.92—11.00 | 10.93 (0.63); 9.75—12.42 |
| Structural racism | 0.03 (0.67); -1.98—1.11 | 0.04 (0.63); -1.98—1.11 |
| Mean caregiver educational attainment | 15.24 (2.36); 3.00—21.00 | 15.29 (2.38); 3.00—21.00 |
| BMI | 19.66 (4.23); 14.57—29.01 | 20.63 (4.36); 14.57—29.01 |
| State income inequality | 0.47 (0.02); 0.43—0.51 | 0.47 (0.02); 0.43—0.51 |
| DHEA (log-transformed and age-residualized) | 0.21 (0.71); -2.69—1.69 | 0.15 (0.77); -2.90—1.79 |
| Testosterone (log-transformed and age-residualized) | 0.17 (0.49); -2.20—1.30 | 0.15 (0.47); -1.44—1.40 |
| Youth-reported PDS (log-transformed and age-residualized) | 0.11 (0.56); -0.68—2.37 | 0.16 (0.55); -0.84—2.41 |
| Caregiver-reported PDS (log-transformed and age-residualized) | 0.23 (0.53); -0.53—2.61 | 0.25 (0.53); -0.73—2.56 |
| **Demographic and Other Study Variables at Baseline and Year 1 for Latinx Girls** | | |
| **Variable** | **Baseline: M(SD); range** | **Year 1: M(SD); range** |
| Age | 9.87 (0.63); 8.92—11.00 | 10.87 (0.65); 9.75—12.25 |
| Structural xenophobia | -0.95 (0.72); -1.75—0.64 | -0.94 (0.72); -1.75—0.64 |
| Mean caregiver educational attainment | 14.47 (3.46); 3.00—21.00 | 14.53 (3.46); 3.00—21.00 |
| BMI | 19.73 (3.98); 14.57—29.01 | 20.81 (4.28); 14.57—29.01 |
| State income inequality | 0.48 (0.02); 0.43—0.51 | 0.48 (0.02); 0.43—0.51 |
| Estradiol (log-transformed and age-residualized) | 0.01 (0.56); -3.00—1.12 | -0.01 (0.58); -2.87—1.17 |
| DHEA (log-transformed and age-residualized) | 0.01 (0.72); -2.78—1.72 | 0.01 (0.74); -3.26—1.58 |
| Testosterone (log-transformed and age-residualized) | -0.05 (0.51); -2.24—1.12 | -0.02 (0.47); -1.82—1.17 |
| Youth-reported PDS (log-transformed and age-residualized) | 0.02 (0.56); -0.85—2.38 | 0.03 (0.59); -1.23—1.93 |
| Caregiver-reported PDS (log-transformed and age-residualized) | 0.02 (0.50); -1.00—2.04 | 0.05 (0.58); -1.39—1.92 |
| **Demographic and Other Study Variables at Baseline and Year 1 for Latinx Boys** | | |
| **Variable** | **Baseline: M(SD); range** | **Year 1: M(SD); range** |
| Age | 9.89 (0.63); 8.92—11.08 | 10.88 (0.64); 9.75—12.25 |
| Structural xenophobia | -0.93 (0.71); -1.75—0.64 | -0.94 (0.72); -1.75—0.64 |
| Mean caregiver educational attainment | 14.66 (3.32); 3.00—21.00 | 14.73 (3.28); 4.00—21.00 |
| BMI | 20.06 (4.12); 14.57—29.01 | 20.98 (4.29); 14.57—29.01 |
| State income inequality | 0.48 (0.02); 0.43—0.51 | 0.48 (0.02); 0.43—0.51 |
| DHEA (log-transformed and age-residualized) | 0.06 (0.74); -2.82—1.68 | 0.04 (0.75); -2.71—1.82 |
| Testosterone (log-transformed and age-residualized) | -0.003 (0.52); -2.69—1.25 | 0.02 (0.51); -2.75—1.24 |
| Youth-reported PDS (log-transformed and age-residualized) | 0.04 (0.54); -0.68—2.37 | 0.10 (0.54); -0.85—2.43 |
| Caregiver-reported PDS (log-transformed and age-residualized) | 0.06 (0.40); -0.53—2.58 | 0.06 (0.44); -0.75—2.38 |
| **Demographic and Other Study Variables at Baseline and Year 1 for Girls** | | |
| **Variable** | **Baseline: M(SD); range or n(%)** | **Year 1: M(SD); range or n(%)** |
| Age | 9.90 (0.62); 8.92—11.00 | 10.91 (0.64); 9.75—12.42 |
| Structural sexism | -0.26 (0.90); -1.85—1.28 | -0.26 (0.91); -1.85—1.28 |
| Mean caregiver educational attainment | 16.34 (2.75); 3.00—21.00 | 16.43 (2.72); 3.00—21.00 |
| BMI | 18.89 (3.85); 14.57—29.01 | 19.74 (4.09); 14.57—29.01 |
| State income inequality | 0.47 (0.02); 0.43—0.51 | 0.47 (0.02); 0.43—0.51 |
| Estradiol (log-transformed and age-residualized) | 0.00 (0.56); -3.41—1.12 | 0.00 (0.58); -3.78—1.17 |
| DHEA (log-transformed and age-residualized) | 0.00 (0.74); -2.89—1.72 | 0.00 (0.73); -3.77—1.67 |
| Testosterone (log-transformed and age-residualized) | 0.00 (0.50); -2.86—1.20 | 0.00 (0.46); -2.10—1.49 |
| Youth-reported PDS (log-transformed and age-residualized) | 0.00 (0.54); -0.86—2.46 | 0.00 (0.58); -1.28—2.10 |
| Caregiver-reported PDS (log-transformed and age-residualized) | 0.00 (0.51); -1.04—2.40 | 0.00 (0.59); -1.51—2.06 |
| Race/ethnicity |  |  |
| Non-Latinx White | 2,887 (51.0%) | 2,810 (52.5%) |
| Asian | 129 (2.3%) | 123 (2.3%) |
| Black | 891 (15.7%) | 793 (14.8%) |
| Latinx | 1,156 (20.4%) | 1,060 (19.8%) |
| Other | 598 (10.6%) | 565 (10.6%) |
| **Differences in Pubertal Development Between Girls and Boys at Baseline** | | |
|  | **Girls: M(SD); range** | **Boys: M(SD); range** |
| DHEA | 72.16 (49.88); 2.68—236.45 | 54.50 (38.56); 1.14—180.62 |
| Testosterone | 35.87 (17.22); 1.86—90.30 | 31.71 (16.10); 0.72—91.86 |
| Youth-reported PDS | 1.69 (0.55); 1.00—4.00 | 1.65 (0.50); 1.00—4.00 |
| Caregiver-reported PDS | 1.77 (0.53); 1.00—4.00 | 1.46 (0.41); 1.00—4.00 |
| **Differences in Pubertal Development Between Girls and Boys at Year 1** | | |
|  | **Girls: M(SD); range** | **Boys: M(SD); range** |
| DHEA | 82.35 (55.06); 1.22—268.83 | 60.55 (43.10); 1.91—231.02 |
| Testosterone | 43.37 (19.86); 4.48—150.54 | 39.40 (19.93); 2.46—118.98 |
| Youth-reported PDS | 1.97 (0.62); 1.00—4.00 | 1.70 (0.51); 1.00—4.00 |
| Caregiver-reported PDS | 2.09 (0.63); 1.00—4.00 | 1.56 (0.45); 1.00—4.00 |

***Note*:** BMI = body-mass index. PDS = Pubertal Development Scale.

**Table S34. Structural Stigma Factor Scores by ABCD Study® State**

| **Structural Stigma Factor Scores by ABCD Study® State** | | | |
| --- | --- | --- | --- |
| **ABCD Study® State** | **Structural Sexism Factor Score** | **Structural Racism Factor Score** | **Structural Xenophobia Factor Score** |
| CA | -0.83 | -0.31 | -1.75 |
| CO | -0.93 | -0.65 | -0.76 |
| CT | -0.57 | -0.33 | -0.59 |
| FL | 0.61 | 0.70 | -0.53 |
| MD | -1.20 | -0.96 | -0.16 |
| MI | -0.15 | 0.20 | -0.28 |
| MN | -0.98 | -0.44 | -0.25 |
| MO | 0.41 | 0.76 | 0.59 |
| NY | -1.19 | -0.68 | -1.20 |
| OK | 0.96 | 0.49 | 0.13 |
| OR | -1.44 | -1.52 | -0.62 |
| PA | -0.05 | 0.40 | 0.39 |
| SC | 1.05 | 1.11 | 0.32 |
| UT | 1.28 | -0.29 | -0.26 |
| VA | -0.37 | -0.37 | -0.49 |
| VT | -1.85 | -1.98 | -0.45 |
| WI | 0.13 | 0.58 | 0.64 |

**Table S35. Structural Stigma Indicators, Data Sources, and Available Years**

| **Structural Racism Indicators, Data Sources, and Available Years** | | |
| --- | --- | --- |
| **Indicator** | **Data Source** | **Available Years** |
| Prompt: On the average (African Americans) have worse jobs, income, and housing than White people. Do you think these differences are: Because most (African Americans) just don’t have the motivation or will power to pull themselves up out of poverty? | General Social Survey | 1977, 1985, 1986, 1988, 1989, 1990, 1991, 1993, 1994, 1996, 1998, 2000, 2002, 2004, 2006, 2008, 2010, 2012, 2014 |
| Prompt: On the average (African Americans) have worse jobs, income, and housing than White people. Do you think these differences are: Mainly due to discrimination?* | General Social Survey | 1977, 1985, 1986, 1988, 1989, 1990, 1991, 1993, 1994, 1996, 1998, 2000, 2002, 2004, 2006, 2008, 2010, 2012, 2014 |
| Prompt: Some people think that (African Americans) have been discriminated against for so long that the government has a special obligation to help improve their living standards. Others believe that the  government should not be giving special treatment to (African Americans). Where would you place yourself on this scale, or haven’t you made up your mind on this? | General Social Survey | 1975, 1983, 1984, 1986, 1987, 1988, 1989, 1990, 1991, 1993, 1994, 1996, 1998, 2000, 2002, 2004, 2006, 2008, 2010, 2012, 2014 |
| Prompt: Are we spending too much, too little, or about the right amount on improving the conditions of Black people?* | General Social Survey | 1973, 1974, 1975, 1976, 1977, 1978, 1980, 1982, 1983, 1984, 1985, 1986, 1987, 1988, 1989, 1990, 1991, 1993, 1994, 1996, 1998, 2000, 2002, 2004, 2006, 2008, 2010, 2012, 2014 |
| Prompt: Now I’m going to ask you about different types of contact with various groups of people. In each situation, please tell me whether you would be very much in favor of it happening, somewhat in favor, neither in favor nor opposed to it happening, somewhat opposed, or very much opposed to it happening: Living in a neighborhood where half of your neighbors were Black. | General Social Survey | 1990, 1996, 1998, 2000, 2002, 2004, 2006, 2008, 2010, 2012, 2014 |
| Prompt: Now I’m going to ask you about different types of contact with various groups of people. In each situation, please tell me whether you would be very much in favor of it happening, somewhat in favor, neither in favor nor opposed to it happening, somewhat opposed, or very much opposed to it happening: Having a close friend or family member marry a Black person. | General Social Survey | 1990, 1996, 1998, 2000, 2002, 2004, 2006, 2008, 2010, 2012, 2014 |
| Prompt: On the average (African Americans) have worse jobs, income, and housing than White people. Do you think these differences are: Because most (African Americans) have less in-born ability to learn? | General Social Survey | 1977, 1985, 1986, 1988, 1989, 1990, 1991, 1993, 1994, 1996, 1998, 2000, 2002, 2004, 2006, 2008, 2010, 2012, 2014 |
| Prompt: Would you vote for a law that says a homeowner can refuse to sell to Black people, or one that says homeowners cannot refuse to sell based on skin color? | General Social Survey | 1977, 1985, 1986, 1988, 1989, 1990, 1991, 1993, 1994, 1996, 1998, 2000, 2002, 2004, 2006, 2008, 2010, 2012, 2014 |
| Prompt: What do you think the chances are these days that a White person won’t get a job or promotion while an equally or less qualified Black person gets one instead? Is this very likely, somewhat likely, or not very likely to happen these days? | General Social Survey | 1990, 1994, 1996, 1998, 2000, 2002, 2004, 2006, 2008, 2010, 2012, 2014 |
| Prompt: Irish people, Italian people, Jewish people, and many other minorities overcame prejudice and worked their way up. Black people should to the same without any special favors. [Rated from 1 (agree strongly) to 5 (disagree strongly).]* | American National Election Studies | 1992, 1994, 1998, 2000, 2004, 2008, 2012, 2016 |
| Prompt: It’s really a matter of some people not trying hard enough. If Black people would only try harder, they could be just as well off as White people. [Rated from 1 (agree strongly) to 5 (disagree strongly).]* | American National Election Studies | 1992; 1994; 2000; 2004; 2008; 2012; 2016 |
| Prompt: If a Black person were put in charge of me, I would not mind taking advice and direction from him or her.* | Project-Implicit: Race | 2002–2017 |
| Prompt: If I had a chance to introduce Black visitors to my friends and neighbors, I would be pleased to do so.* | Project-Implicit: Race | 2002–2017 |
| Prompt: I would rather not have Black people live in the same apartment building I live in. | Project-Implicit: Race | 2002–2017 |
| Prompt: I would probably feel somewhat self-conscious dancing with a Black person in a public place. | Project-Implicit: Race | 2002–2017 |
| Prompt: I would not mind at all if a Black family with about the same income and education as me moved in next door.* | Project-Implicit: Race | 2002–2017 |
| Prompt: I think that Black people look more similar to each other than White people do. | Project-Implicit: Race | 2002–2017 |
| Prompt: Interracial marriage should be discouraged to avoid the “who-am-I?” confusion that the children feel. | Project-Implicit: Race | 2002–2017 |
| Prompt: I get very upset when I hear a White person make a prejudicial remark about Black people.* | Project-Implicit: Race | 2002–2017 |
| Prompt: I favor open housing laws that allow more racial integration of neighborhoods.* | Project-Implicit: Race | 2002–2017 |
| Prompt: It would not bother me if my new roommate was Black.* | Project-Implicit: Race | 2002–2017 |
| Prompt: It is likely that Black people will bring violence to neighborhoods when they move in. | Project-Implicit: Race | 2002–2017 |
| Prompt: The federal government should take decisive steps to override the injustices Black people suffer at the hands of local authorities.* | Project-Implicit: Race | 2002–2017 |
| Prompt: Black and White people are inherently equal.* | Project-Implicit: Race | 2002–2017 |
| Prompt: Black people are demanding too much too fast in their push for equal rights. | Project-Implicit: Race | 2002–2017 |
| Prompt: White people should support Black people in their struggle against discrimination and segregation.* | Project-Implicit: Race | 2002–2017 |
| Prompt: Generally, Black people are not as smart as White people. | Project-Implicit: Race | 2002–2017 |
| Prompt: I worry that in the next few years I may be denied my application for a job or a promotion because of preferential treatment given to minority group members. | Project-Implicit: Race | 2002–2017 |
| Prompt: Some Black people are so touchy about race that it is difficult to get along with them. | Project-Implicit: Race | 2002–2017 |
| Prompt: Do Black people tend to be violence prone, or do they tend not to be prone to violence?* | Project-Implicit: Race | 2002–2017 |
| Prompt: Do Black people tend to prefer to be self-supporting or do they tend to prefer to live off welfare? | Project-Implicit: Race | 2002–2017 |
| **Structural Xenophobia Indicators, Data Sources, and Available Years** | | |
| **Indicator** | **Data Source** | **Available Years** |
| Feelings thermometer towards illegal immigrants.* | American National Election Studies | 2004, 2008, 2012, 2016 |
| Feelings thermometer towards Hispanic people.* | American National Election Studies | 1996, 2000, 2002, 2004, 2008, 2012, 2016 |
| Composite index of supportive (vs. restrictive) nature of states laws/policies on the following domains: (1) access to health services for immigrants, (2) state or local law enforcement cooperation with federal immigration enforcement, (3) use of non-English language, (4) immigrant employment in a broad segment of the private sector, (5) immigrant access to a broad class of business licenses, (6) access to rental housing for immigrants, (7) nondiscrimination based on citizenship or immigration status, (8) access relevant services or opportunities without identification, (9) access to higher education for immigrants, and (10) access to driver’s licenses for immigrants.* | Wake Forest University | 2016 |
| **Structural Sexism Indicators, Data Sources, and Available Years** | | |
| **Indicator** | **Data Source** | **Available Years** |
| Prompt: Tell me if you agree or disagree with this statement: “Most men are better suited emotionally for politics than are most women.” | General Social Survey | 1974, 1975, 1977, 1978, 1982, 1983, 1985, 1986, 1988, 1989, 1990, 1991, 1993, 1994, 1996, 1998, 2000, 2002, 2004, 2006, 2008, 2010, 2012, 2014 |
| Prompt: Tell me if you agree or disagree with this statement: “It is much better for everyone involved if the man is the achiever outside the home and the woman takes care of the home and family.” | General Social Survey | 1977, 1985, 1986, 1988, 1989, 1990, 1991, 1993, 1994, 1996, 1998, 2000, 2002, 2004, 2006, 2008, 2010, 2012, 2014 |
| Percent of women who live in a county without an abortion provider. | Guttmacher Institute | 2014 |
| Ratio of men’s to women’s median usual weekly earnings of full-time wage and salary workers. | Bureau of Labor Statistics | 2016 |
| Employment and earnings composite index of women’s status related to occupation including four components: (1) women’s median annual earnings, (2) ratio of women’s to men’s earnings, (3) women’s labor force participation, and (4) women’s representation in managerial and professional occupations.* | Bureau of Labor Statistics | 2013 |
| Ratio of men’s to women’s labor force participation rates, ages 16+. | Current Population Survey | 2016 |
| Political composite index reflecting four components: (1) women’s voter registration, (2) women’s voter turnout, (3) women’s representation in elected office, and (4) the existence of institutional resources for women.* | Center for American Women in Politics | 2015 |
| Social and economic autonomy composite index including four components: (1) proportion of women with health insurance, (2) proportion of women with a college degree, (3) women’s business ownership, and (4) proportion of women living above the federal poverty level.* | Integrated Public Use Microdata Series | 2013 |
| Prompt: Women hold a smaller portion of the science and engineering faculty positions at top research universities than do men. Please rate how important you think the following factor is for explaining this difference: “On average, whether consciously or unconsciously, men are favored in hiring and promotion.”* | Project Implicit: Gender-Science | 2003–2018 |
| Prompt: Women hold a smaller portion of the science and engineering faculty positions at top research universities than do men. Please rate how important you think the following factor is for explaining this difference: “Directly or indirectly, boys and girls tend to receive different levels of encouragement for developing scientific interest.”* | Project Implicit: Gender-Science | 2003–2018 |
| Prompt: Women hold a smaller portion of the science and engineering faculty positions at top research universities than do men. Please rate how important you think the following factor is for explaining this difference: “Different proportions of men and women are found among people with the very highest levels of math ability.” | Project Implicit: Gender-Science | 2003–2018 |
| Prompt: Women hold a smaller portion of the science and engineering faculty positions at top research universities than do men. Please rate how important you think the following factor is for explaining this difference: “On average, men and women differ in their willingness to spend time away from their families.” | Project Implicit: Gender-Science | 2003–2018 |
| Prompt: Women hold a smaller portion of the science and engineering faculty positions at top research universities than do men. Please rate how important you think the following factor is for explaining this difference: “On average, men and women differ in their willingness to devote the time required by such ‘high-powered’ positions.” | Project Implicit: Gender-Science | 2003–2018 |
| Prompt: Women hold a smaller portion of the science and engineering faculty positions at top research universities than do men. Please rate how important you think the following factor is for explaining this difference: “On average, men and women differ naturally in their scientific interest.” | Project Implicit: Gender-Science | 2003–2018 |
| Prompt: Suppose that ten men at a typical U.S. university were picked at random. How many would you predict will graduate with a scientific major (science, technology, engineering, or mathematics)? | Project Implicit: Gender-Science | 2003–2018 |
| Gender-Science Implicit Association Test (IAT) | Project Implicit: Gender-Science | 2003–2018 |
| Prompt: How strongly do you associate career and family with males and females? | Project Implicit: Gender-Career | 2005–2018 |
| Gender-Career Implicit Association Test (IAT) | Project Implicit: Gender-Career | 2005–2018 |

*Reverse scored such that higher ratings represented higher levels of structural stigma.
